# 3D Printing Plasmonic-Enhanced Sulfurized Polyacrylonitrile Cathodes for High-Energy Li–S Microbatteries

**DOI:** 10.1007/s40820-026-02204-w

**Published:** 2026-05-04

**Authors:** Yu Liu, Penghao Fu, Jieshan Qiu, Zhiyu Wang

**Affiliations:** 1 State Key Lab of Fine Chemicals, Frontiers Science Center for Smart Materials Oriented Chemical Engineering, Liaoning Key Lab for Energy Materials and Chemical Engineering, School of Chemical Engineering, Dalian University of Technology, Dalian, 116024 People’s Republic of China; 2 School of Chemical Engineering and Technology, Xinjiang University, Urumqi, 830046 People’s Republic of China; 3 College of Chemical Engineering, Beijing University of Chemical Technology, Beijing, 100029 People’s Republic of China

**Keywords:** Quasi-solid-state battery, Li–S microbattery, Plasmonic enhancement

## Abstract

The rapid expansion of the Internet of Things (IoT) has fueled the demand for high-energy, compact microbatteries capable of powering energy-demanding IoT devices in small, flexible formats. Li–S batteries offer a promising solution but suffer from more intense polysulfide (LiPS) shuttling in the limited volume of microbatteries. Here, we achieved a high-energy quasi-solid-state Li–S microbattery by employing 3D-printed hierarchically structured sulfurized polyacrylonitrile (3D-HSPAN) cathodes with plasmonic enhancement. The direct ink writing technique produces shape-customizable 3D-HSPAN cathodes with precise architectural engineering, ultra-high mass loading up to 37.1 mg cm^−2^, and greatly improved ionic transport. Plasmonic MXene is harnessed to further boost LiPS-free redox conversion through synergistic photothermal effect and hot-carrier injection under near-infrared irradiation. Paired with a LiNO_3_ sustained-release carbonate-based gel polymer electrolyte, such quasi-solid-state Li–S microbatteries deliver high areal capacities over 18.1 mAh cm^−2^ and exceptional areal energy density reaching 30.7 mWh cm^−2^. Their versatility in flexible, transparent, and shape-customizable formats is demonstrated for wearable electronics and low-temperature operation. This work establishes a framework for uniting additive manufacturing, high-energy redox chemistry, and light-harvesting strategies to advance energy solutions.

**Supplementary Information:**

The online version contains supplementary material available at 10.1007/s40820-026-02204-w.

## Highlights

- The DIW technique fabricates shape-customizable 3D-printed hierarchically structured sulfurized polyacrylonitrile (3D-HSPAN) cathodes with precisely controlled scaffolds and ultra-high mass loading up to 37.1 mg cm^−2^.
- Plasmonic MXene further regulates the redox kinetics of solid-state sulfur chemistry for 3D-HSPAN cathode via synergistic photothermal effect and hot-carrier injection.
- The quasi-solid-state Li–S microbattery delivers an exceptional areal capacity of 18.1 mAh cm^−2^ and a high areal energy density of 30.7 mWh cm^−2^.

**Supplementary Information:**

The online version contains supplementary material available at 10.1007/s40820-026-02204-w.

## Graphical abstract

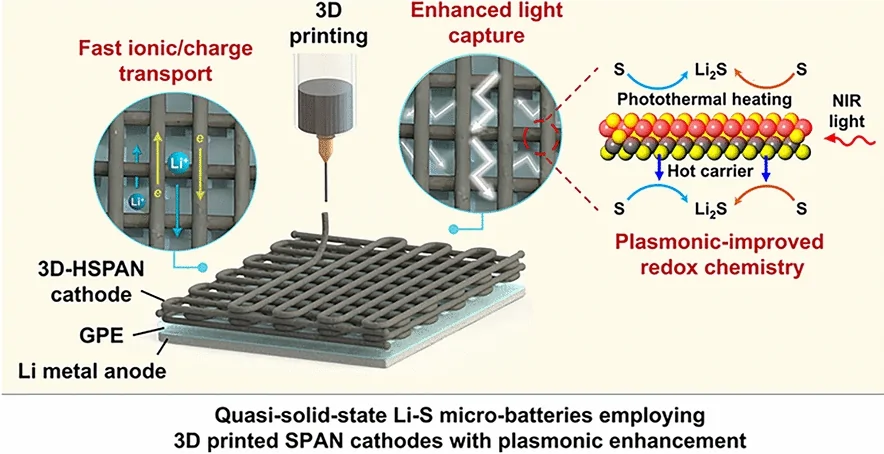

## Introduction

Rapid expansion of the Internet of Things (IoT) is reshaping the global energy landscape, with IoT devices projected to reach one trillion by 2035 [1, 2]. Unlike conventional consumer electronics, IoT devices, including wearable devices, wireless sensors, implantable medical devices, and soft robotics, demand rechargeable microbatteries to utilize high energy within compact and flexible geometries [3]. The limited energy density of conventional Li-ion batteries (LIBs) restricts their applicability to power-demanding IoT platforms, particularly when scaled down. Benefiting from high theoretical energy density (2600 Wh kg^−1^), Li–S batteries offer an attractive alternative to address this issue [4]. Nevertheless, translating Li–S chemistry into microbatteries faces fundamental challenges arising from its solid–liquid–solid redox pathways with soluble polysulfides (LiPS) as redox intermediates (*i.e.*, S_(*s*)_ + Li^+^  ↔ LiPS_(*l*)_ ↔ Li_2_S_(*s*)_). Within the limited volume of microbatteries, the dissolution of a small amount of LiPS rapidly saturates the electrolyte, which hinders efficient sulfur conversion, accelerates the LiPS shuttling effect, and restricts ionic transport, thereby degrading cell performance [5]. The solid–liquid–solid redox mechanism is also highly temperature sensitive. Elevated temperatures exacerbate the LiPS shuttling, while low temperatures slow redox kinetics. The small dimension of microscale batteries magnifies these instabilities, complicating the integration of temperature-sensitive Li–S chemistry. In addition, the conventional slurry-casting approach to electrode fabrication is unsuitable to address the severe mechanical stress associated with over 80% volume expansion of sulfur upon cycling, particularly within the constrained space of microbatteries [6]. On the anode side, LiPS-saturated electrolyte aggravates Li metal corrosion and dendrite growth [7]. These problems further damage cell performance and safety, raising substantial obstacles to the practical realization of Li–S microbatteries.

Sulfurized polyacrylonitrile (SPAN) represents a class of effective cathode materials to overcome LiPS challenges due to their LiPS-free solid–solid redox route, enabled by covalently binding short-chain sulfur species (*e.g.*, S, S_2_, S_3_) within a conductive polymer matrix [8, 9]. This mechanism avoids LiPS shuttling and reduces the temperature dependence of Li–S redox kinetics, resulting in superior cycling stability and operational adaptability. Moreover, SPAN cathode delivers high capacities exceeding 800 mAh g^−1^, holding the potential to maximize areal capacity in microbatteries [10]. Nevertheless, SPAN cathodes generally suffer from rapid capacity decay at high mass loading owing to their intrinsic low conductivity, which is hardly addressed by the conventional way of slurry-casting electrode manufacturing. 3D printing offers a viable solution to this issue via programmable, geometry-tailored electrode fabrication with superior design flexibility [11]. In particular, direct ink writing (DIW) excels in high-resolution, programmable deposition of functional inks across diverse material systems. It allows the production of complex 3D architected electrodes with precise control over geometries, porosity, spatial organization, and mechanical properties [12, 13]. These optimizations enhance ionic transport, reduce diffusion difficulties, and accommodate cycling-induced stress in thick electrodes with less trade-off in electrochemical accessibility [14]. This technique has been applied to micro-supercapacitors and micro-LIBs (*e.g.*, LiFePO_4_||Li_4_Ti_5_O_12_), typically achieving areal capacities below 3–5 mAh cm^−2^ and limited energy densities (< 5–10 mWh cm^−2^) [15–17]. The integration of high-capacity SPAN with 3D printing to realize high-energy microbatteries also remains largely unexplored.

**Fig. 1:**
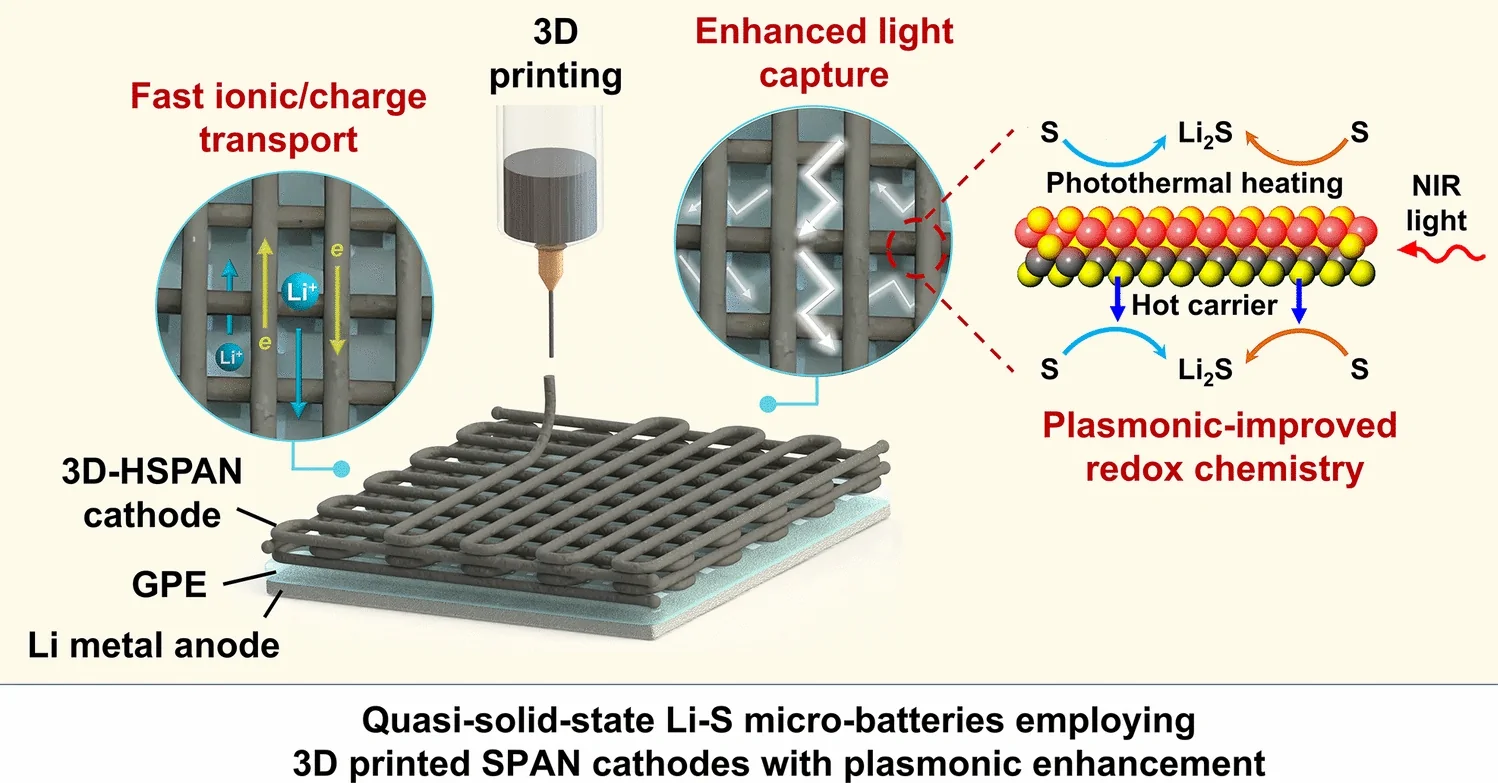
Schematic illustration of the configuration and advantages of a quasi-solid-state Li–S microbattery employing a 3D-printed HSPAN cathode with plasmonic enhancement

Beyond engineering electrode architecture, light-assisted strategies have emerged as another effective way to enhance battery performance [18]. Employing plasmonic materials has demonstrated effectiveness in enhancing sulfur utilization in Li–S batteries, lowering charge-transfer resistance in LIBs, and even achieving partially photo-chargeable cells [19, 20]. Specifically, MXenes provide a compelling plasmonic platform for improving Li–S redox chemistry because of their prolonged hot-carrier lifetimes compared to noble metals, attributed to extended electronic mean free paths arising from a nearly fourfold lower carrier density [21–23]. Compared to wide-bandgap semiconductors, MXenes sustain efficient hot-carrier generation as a result of high carrier densities and efficient absorption across the visible to infrared spectrum [24]. Furthermore, they have been extensively recognized for their sulfur tolerance, high conductivity, and strong chemical interactions for stabilizing polysulfides [22]. Nevertheless, most light-assisted batteries rely on liquid electrolytes, suffering from limited stability and safety, while utilizing slurry-casting electrodes with restricted light capture efficiency. These limitations could be overcome in 3D-printed electrodes with optimized light capture within ordered optical transmission pathways. Leveraging this advantage, we designed a high-energy quasi-solid-state Li–S microbattery by harnessing 3D-printed hierarchical SPAN cathodes (3D-HSPAN) with plasmonic enhancement (Fig. 1). The DIW technique fabricates shape-customizable 3D-HSPAN cathodes with highly ordered, hierarchically open porous architectures, reduced tortuosity, and embedded plasmonic functionality. This structural design decouples areal loading from transport resistance, enabling ultra-high mass loadings up to 37.1 mg cm⁻^2^ while maintaining rapid reaction kinetics in thick electrodes. MXene further acts as a plasmonic enhancer, enabling photothermal heating and hot-carrier injection for regulating the redox kinetics of solid-state sulfur chemistry for the 3D-HSPAN cathode. This 3D-HSPAN cathode is paired with a carbonate-based gel polymer electrolyte (GPE) with sustained LiNO_3_ release, yielding a quasi-solid-state microbattery with an exceptional areal capacity of 18.1 mAh cm^−2^ and record-high areal energy densities (30.7 mWh cm⁻^2^). Transparent quasi-solid-state Li–S microbattery with customized shape is further assembled to demonstrate the plasmonic enhancement in capacity loading and low-temperature operation.

## Experimental Section

### Synthesis of HSPAN

The HPAN was first synthesized by radical polymerization of acrylonitrile (AN) in acetone (1:2 in volume ratio) with 2,2-azobisisobutyronitrile (AIBN, 0.5 mg mL^−1^) as the initiator at 70 °C for 2 h in Ar. The HSPAN was prepared via a melt-infusion method. Specifically, the HPAN was mixed with sublimed sulfur in a mass ratio of 1:4 through ball milling, followed by annealing at 350 °C for 6 h and then at 200 °C for 5 h in Ar flow.

### Preparation of GPE

A LiNO_3_-loaded PVDF-HFP film was first fabricated by dispersing LiNO_3_ (0.2 g) and poly(vinylidene fluoride-co-hexafluoropropylene) (PVDF-HFP, 0.4 g) in acetone (30 mL) under stirring for 2 h at 90 °C, followed by casting onto a quartz plate and vacuum drying at 50 °C for 12 h. The formed film was peeled off and immersed in 1.0 M LiPF_6_ in ethylene carbonate (EC) and diethyl carbonate (DEC) (1:1 by volume) with fluoroethylene carbonate (FEC, 10 wt.%), pentaerythritol tetraacrylate (PETEA, 1.0 wt%) and AIBN (0.1 wt%) for 12 h in a glove box, followed by heating at 60 °C for 3 h.

### Preparation of Direct Ink Writing Ink and 3D-HSPAN Electrode

The DIW ink was prepared by dispersing carboxymethyl cellulose (CMC, 0.1 g), HSPAN (0.6 g), graphene (0.25 g), and Ti_3_C_2_T_*x*_ MXene (0.05 g) in DI water (5 mL) under stirring for 2 h at 80 °C. The 3D printing was conducted on an air-powered fluid dispenser equipped with a three-axis motion system. The DIW ink was transferred into a syringe with a nozzle diameter of 320 μm. The electrode was printed layer by layer to the designed pattern at a pressure of 25 psi at a rate of 5 mm s^–1^, yielding a single-layer thickness of *ca.* 0.6 mm. After printing, the resultant architectures were freeze-dried to yield 3D-HSPAN electrodes.

### Assembly and Tests of Symmetric Li|GPE|Li Cells and Asymmetric Li|GPE|Cu Cells

The symmetric Li||Li cells were assembled by using Li metal foil as both the working and counter electrodes in GPE or LE. They were cycled at a capacity of 1 mAh cm^–2^ and a current density of 1.0 mA cm^–2^ for Li plating/stripping. The asymmetric Li||Cu cells were assembled by using Li metal foil against Cu foil using GPE or LE. The coulombic efficiency (CE) tests were conducted at a current density of 0.5 mA cm^–2^ with a cycling capacity of 0.5 mAh cm^–2^.

### Assembly and Tests of Quasi-Solid-State 3D-HSPAN|GPE|Li Cells

The quasi-solid-state 3D-HSPAN|GPE|Li cells were assembled using a 3D-HSPAN cathode against Li metal foil in GPE with a polypropylene–polyethylene (PP) separator. Galvanostatic tests were performed on Land CT3001A battery testers. The cells were cycled between 1.0–3.0 V (vs. Li/Li^+^) at various current rates. The rate capability was evaluated at various current rates of 0.05–0.3 C. The capacities were calculated based on sulfur mass. For comparison, the 3D-HSPAN|LE|Li coin cells were also assembled and tested similarly, except that GPE was replaced with LE. Electrochemical impedance spectroscopy (EIS) and cyclic voltammetry (CV) tests were performed on a CHI 760E electrochemical workstation. The EIS spectra were recorded in a frequency range of 100 kHz–0.01 Hz with an amplitude of 5 mV. The CV tests were performed from 1.0–3.0 V (vs. Li/Li^+^) at a scan rate of 0.1 mV s^−1^.

Transparent quasi-solid-state 3D-HSPAN|GPE|μLi microbatteries were assembled by stacking a 3D-HSPAN cathode, GPE film, and ultra-thin Li foil (20 μm in thickness). The cells were encapsulated within transparent polyethylene terephthalate (PET) film. The galvanostatic discharge–charge tests were carried out between 1.0–3.0 V (vs. Li/Li^+^) at various current rates. The Xe lamp (CEL-PE300L-3A) was employed to simulate sunlight exposure, with a power density of 100 mW cm^−2^.

## Results and Discussion

### Fabrication and Characterization of 3D-HSPAN Cathode

**Fig. 2:**
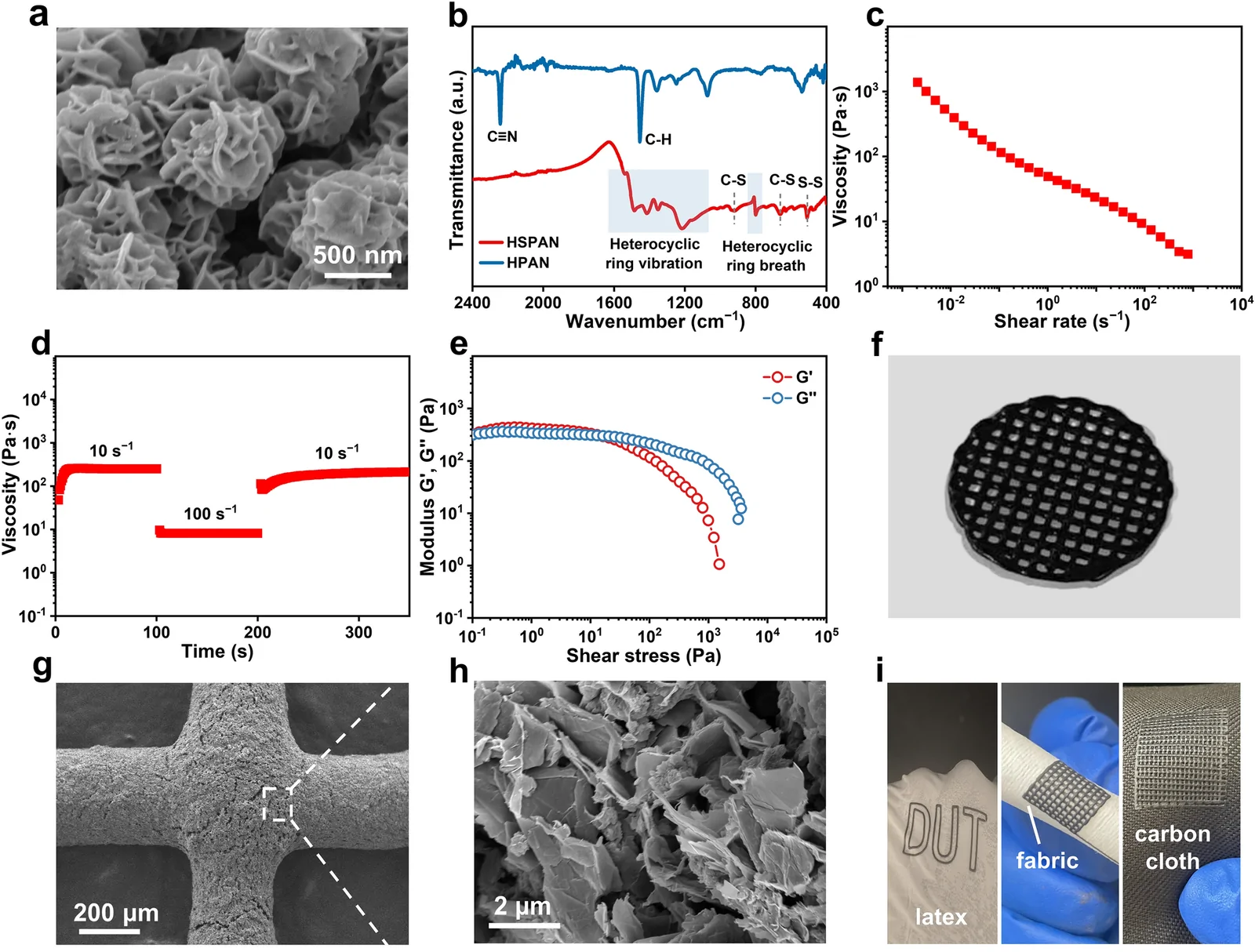
**a** SEM image of HSPAN. **b** FT-IR spectra of HPAN and HSPAN. **c** Variation of apparent viscosity with shear rate for the aqueous ink composed of HSPAN, MXene, graphene, and CMC. **d** Viscosity change of this printable ink over time at different shear rates. **e** Dynamic modulus of this ink as a function of shear stress. **f** Optical image of a 3D-HSPAN electrode. **g, h** SEM images of the grid structure in the 3D-HSPAN electrode. **i** Optical images of 3D-HSPAN electrodes printed on various substrate materials

Hierarchical polyacrylonitrile (HPAN) is first obtained by radical polymerization of acrylonitrile (AN), initiated by 2,2-azobisisobutyronitrile (AIBN) (Fig. S1). Subsequent annealing with sulfur at 350 °C in an Ar atmosphere induces dehydrogenation and cyclization of PAN chains, accompanied by sulfur incorporation. This process yields hierarchically structured sulfurized PAN (HSPAN) composed of loosely stacked nanosheets with an average thickness of 20–30 nm (Fig. 2a). X-ray diffraction (XRD) analysis shows that HSPAN forms only at annealing temperatures above 350 °C (Fig. S2a). As the temperature increases from 350 to 550 °C, sulfur content decreases from 42 to 28 wt%, indicating substantial sulfur loss during high-temperature treatment (Fig. S2b). Fourier-transform infrared (FT-IR) spectra confirm this sulfur loss via the cleavage of C–S bonds in samples annealed above 450 °C (Fig. S2c). Elemental mapping visualizes uniform sulfur distribution alongside C and N elements throughout HSPAN prepared under optimal conditions (Figs. S2d and S3). FT-IR spectra suggest that sulfur is bonded to the PAN rings through C–S bonds (660 and 919 cm^−1^). Meanwhile, the disappearance of the C≡N stretching vibration (2244 cm^−1^), together with the rise of vibrational bands at 1221–1541 cm^−1^ and breathing modes near 800 cm^−1^ for heterocyclic rings, confirms the formation of cyclized PAN rings (Fig. 2b) [25]. The presence of sulfur species is further verified by the S 2*p* XPS spectrum, displaying the doublets at 161.6/162.9 eV for CS_*x*_H species and 163.5/164.7 eV for S–S/C–S bonds, together with signals at 285.7 eV (C–S bond) and 288.4 eV (S–C=N bond) in C 1*s* spectrum (Fig. S4) [8, 26]. Elemental analysis determines a sulfur content of *ca.* 42 wt% in HSPAN.

The HSPAN is formulated into a printable ink by blending with MXene, graphene, and carboxymethyl cellulose (CMC) binder in deionized water for DIW. This composite ink enables 3D printing of electrode architectures with high active material utilization while maintaining efficient ionic diffusion and charge transport. Rheological measurements indicate its pronounced shear-thinning behavior, as viscosity decreases sharply with increasing shear rate (Fig. 2c). Peak-hold-step (PHS) tests further reveal a rapid and fully reversible viscosity response. The viscosity drops abruptly at a fast shear rate of 100 s^**–**1^, followed by complete recovery once the rate returns to 10 s^**–**1^ (Fig. 2d). Stress-sweep-step (SSS) analysis shows a solid-like response under pressures below 16 Pa, with the storage modulus (G’) exceeding the loss modulus (G”). This elastic dominance ensures structural integrity, printing precision, and mechanical stability during fabrication and utilization of the electrode (Fig. 2e) [27]. These rheological benefits support DIW of shape-customizable electrodes with precisely tailored porosity, tortuosity, thickness, and mass loading. As a demonstration, a 3D-HSPAN electrode, featuring an ordered macroporous framework with a grid width of 320 μm and a thickness of 0.6 mm, is fabricated to achieve an active mass loading of 8.9 mg cm^**−**2^ (Fig. 2f, g). The printed electrode grid is constructed from uniformly dispersed MXene nanosheets interwoven with graphene networks (Fig. 2h). They form a highly conductive and mesoporous framework within the ordered macroporous scaffold of 3D-HSPAN. The interwoven MXene and graphene enlarge the accessible surface area of 3D-HSPAN (50.4 m^2^ g^−1^), 1.8 times larger than that of the slurry-casting HSPAN electrode (SC-HSPAN, 28 m^2^ g^−1^). This hierarchically porous architecture promotes electrolyte infiltration, facilitates ionic diffusion, and ensures charge transport over long ranges. Therefore, it is capable of overcoming diffusion limitation and high resistance commonly observed in thick slurry-casting electrodes. Moreover, DIW enables facile control over active mass loading of the electrode through layer-by-layer printing. For example, successive printing of four to ten layers increases the active mass loading of 3D-HSPAN electrodes to 16.05–37.1 mg cm^**–**2^, respectively. By adjusting ink rheology or printing program, on-demand construction of 3D-HSPAN electrodes with diverse geometries could also be achieved on various substrates (*e.g.*, latex, fabric, and carbon cloth) for application-specific requirements (Fig. 2i).

### Preparation and Characterization of GPE

A carbonate-based GPE is designed for a 3D-HSPAN cathode by impregnating a LiNO_3_-loaded poly(vinylidene fluoride-co-hexafluoropropylene)(PVDF-HFP) membrane with a liquid electrolyte (LE) in the presence of pentaerythritol tetraacrylate (PETEA) and AIBN. The LE consists of 1.0 M LiPF_6_ in ethylene carbonate (EC) and diethyl carbonate (DEC) with fluoroethylene carbonate (FEC, 10 wt%) additive. The LiNO_3_-loaded PVDF-HFP membrane exhibits a highly porous and interconnected framework, with uniformly distributed micrometer-scale voids. This well-defined architecture facilitates efficient electrolyte uptake and promotes ion transport. Elemental mapping reveals a homogeneous distribution of F, O, and N elements across the membrane, verifying uniform incorporation of LiNO_3_ within the polymer matrix (Fig. S5). It is utilized to generate a Li_3_N-rich solid electrolyte interphase (SEI) for suppressing Li dendrite growth upon cycling. The AIBN initiates radical polymerization of PETEA at 60 °C by attacking its terminal C=C bonds, yielding a crosslinked polymeric network that immobilizes the LE within the PVDF-HFP film (Fig. S6a, b) [28]. Successful polymerization of PETEA is verified by the disappearance of the stretching bands of terminal C=C groups at 1626 cm^–1^ in FT-IR spectra (Fig. S6c). The resulting GPE achieves an ionic conductivity (*σ*) of 0.33 mS cm^−1^ at 30 °C, which increases from 0.15 to 0.56 mS cm^−1^ as the temperature rises from −20 to 60 °C (Fig. S7a). Both the GPE and LE exhibit a linear ln*σ*^−1^ − 1/*T* relationship according to the Arrhenius transport model [29]. The GPE displays a conduction activation energy (*E*_a, GPE_ = 11.13 kJ mol^−1^) comparable to that of the LE (*E*_a, LE_ = 10.43 kJ mol^−1^). It also exhibits an improved Li^+^ transference number (*t*_Li⁺_ = 0.53) relative to LE (*t*_Li⁺_ = 0.42), attributed to the PETEA backbone, where electron-deficient C=O groups immobilize anions and facilitate preferential Li⁺ migration (Fig. S7b–d). These improvements enable superior interfacial kinetics, evidenced by a higher exchange current density (*j*_0_ = 9.40 mA cm^−2^) in GPE compared to LE (*j*_0_ = 7.84 mA cm^−2^) in Li||Li symmetric cells (Fig. S8). Furthermore, confinement of LE within immobile polymer matrix suppresses solvent mobility and parasitic reactions at the electrode interface, thereby extending the electrochemical stability window (ESW) of GPE beyond 4.5 V, exceeding the 4.2 V limit of LE (Fig. S9).

### Performance of Quasi-Solid-State 3D-HSPAN|GPE|Li Microbatteries

**Fig. 3:**
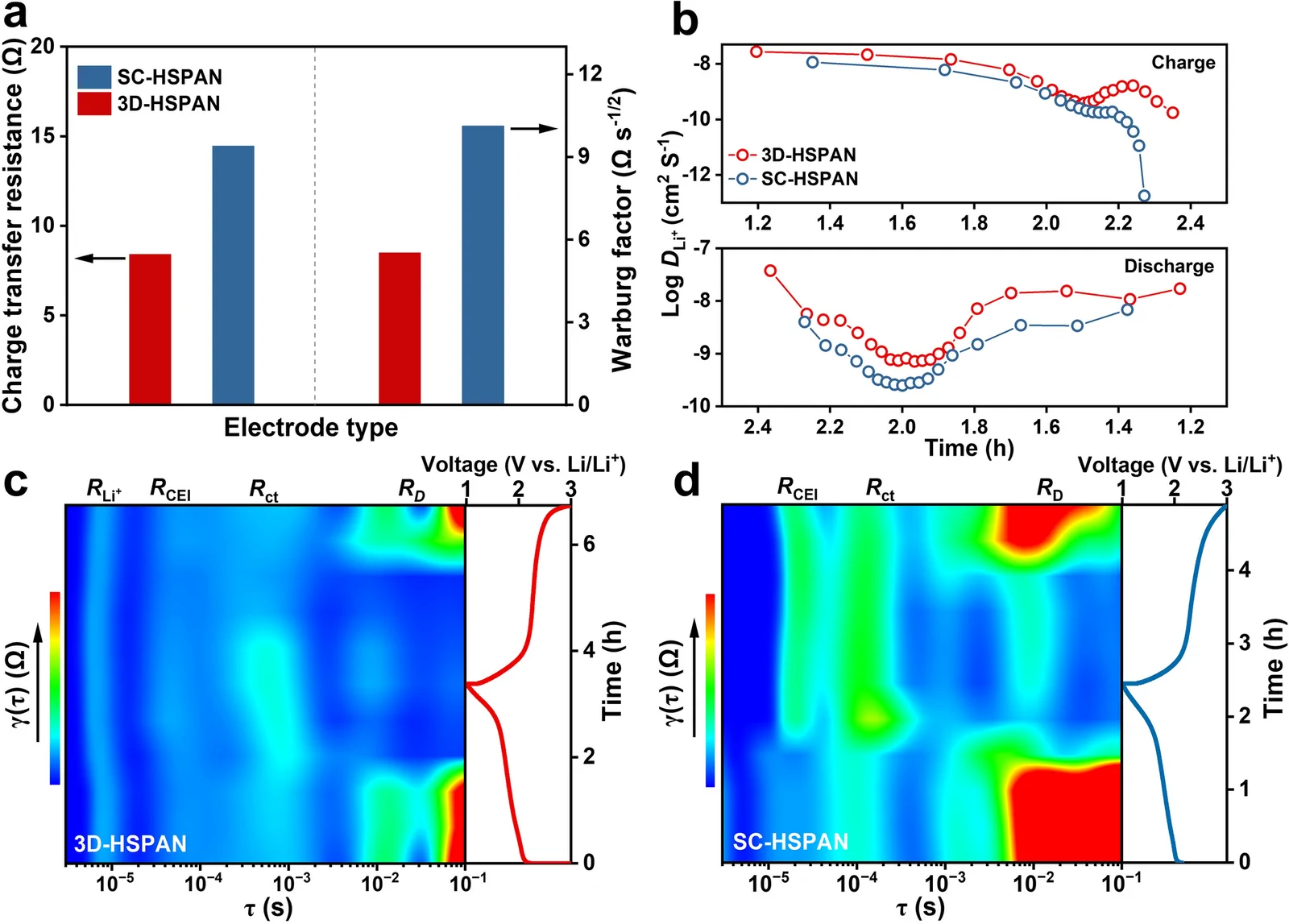
**a** A comparison of 3D-HSPAN and SC-HSPAN cathodes in charge-transfer resistance and Warburg factor in carbonate-based GPE. **b** Li⁺ diffusion coefficients determined from GITT analysis for 3D-HSPAN and SC-HSPAN cathodes during cycling in GPE. DRT profiles derived from in situ EIS spectra of **c** 3D-HSPAN and **d** SC-HSPAN cathodes in GPE

The 3D-HSPAN electrode displays a highly ordered, hierarchically open porous framework with reduced tortuosity, facilitating fast ionic diffusion and redox kinetics even in thick electrodes. Electrochemical impedance spectroscopy (EIS) reveals a substantial reduction in charge-transfer resistance (*R*_ct_ = 8.41 Ω) and Warburg factor (*δ* = 5.52 Ω s^−1/2^) for 3D-HSPAN relative to SC-HSPAN (*R*_ct_ = 15.45 Ω, δ = 10.13 Ω s^−1/2^) in carbonate-based GPE (Figs. 3a and S10). This translates to a more than 3.37-fold enhancement in Li^+^ diffusion rate in 3D-HSPAN, given that the Li^+^ diffusion coefficient (*D*_Li⁺_) scales inversely with *δ*^2^ as *D*_Li⁺_ = *R*^2^*T*^2^/2*A*^2^*n*^4^*F*^4^*C*^2^*δ*^2^, where *R* is the gas constant, *T* is the absolute temperature, *A* is the area of the electrode, *n* is the electron number, *F* is the Faraday constant, and *C* is the Li^+^ concentration [30]. Galvanostatic intermittent titration technique (GITT) validates a 3.63 times greater *D*_Li⁺_ for 3D-HSPAN than in SC-HSPAN (Figs. 3b and S11). To decouple overlapping impedance responses, distributed relaxation time (DRT) analysis is derived from in situ EIS for electrodes with different architectures in GPE (Figs. 3c, d and S12) [31]. Three distinct peaks appear at different time constants *τ* of 10^−5^–10^−4^, 10^−4^–10^−3^, and 10^−2^–10^−1 ^s. As the absence of MXene largely reduces the electrode conductivity, resulting in pronounced peak increase at *τ* of 10^−4^–10^−3^s, which are assigned to *R*_ct_ (Fig. S13). The DRT analysis is further performed on 3D-HSPAN electrodes with varying mass loadings. As the mass loading increases, the peak at *τ* of 10^–2^–10^–1^s exhibits the most significant amplification, reflecting the growth of ionic diffusion resistance (*R*_D_) in thicker electrodes (Fig. S14a). On this basis, the attribution of resistance (*R*_CEI_) of cathode–electrolyte interphase (CEI) is verified by the intensified peak at *τ* of 10^−5^–10^−4^s for 3D-HSPAN operating in GPE lacking FEC and LiNO_3_, where the CEI exhibits reduced ionic conductivity due to the presence of fewer inorganic species (Fig. S14b). Upon cycling, SC-HSPAN undergoes a progressive and irreversible rise in* R*_CEI_ and *R*_ct_, indicative of continuous degradation of CEI and charge-transfer kinetics, respectively. In contrast, the 3D-HSPAN electrode maintains low *R*_CEI_ and *R*_ct_ throughout cycling, owing to superior interfacial stability and conductivity. Notably, 3D-HSPAN also exhibits markedly lower *R*_D_, manifesting the benefits of 3D-printed electrode architectures in promoting mass transport. Additional relaxation signals exclusively appear below 10^−5^s for 3D-HSPAN, reflecting ultra-fast ionic diffusion and interfacial transport within its hierarchical macro-mesoporous framework with high conductivity and reduced tortuosity [32, 33]. Complementary distribution of diffusion time (DDT) analysis offers further insight into diffusive behaviors across timescales (Fig. S15) [31]. The SC-HSPAN exhibits a dominant, narrow peak at* τ* = 20.4 s, showing slow and bottlenecked diffusion due to poor electrolyte infiltration in a dense electrode with high tortuosity. In contrast, 3D-HSPAN features a much lower and broader peak at* τ* = 16.8s, together with weaker secondary features, reflecting more distributed transport channels and smoother mass diffusion across its hierarchical framework. This reduced diffusion resistance translates into superior ionic accessibility and diminished diffusion-limited polarization, improving rate capability and cell life. Collectively, these findings highlight the effectiveness of 3D printing in accelerating both charge transfer and ionic transport in HSPAN-based electrodes beyond the common slurry-casting technique.

**Fig. 4:**
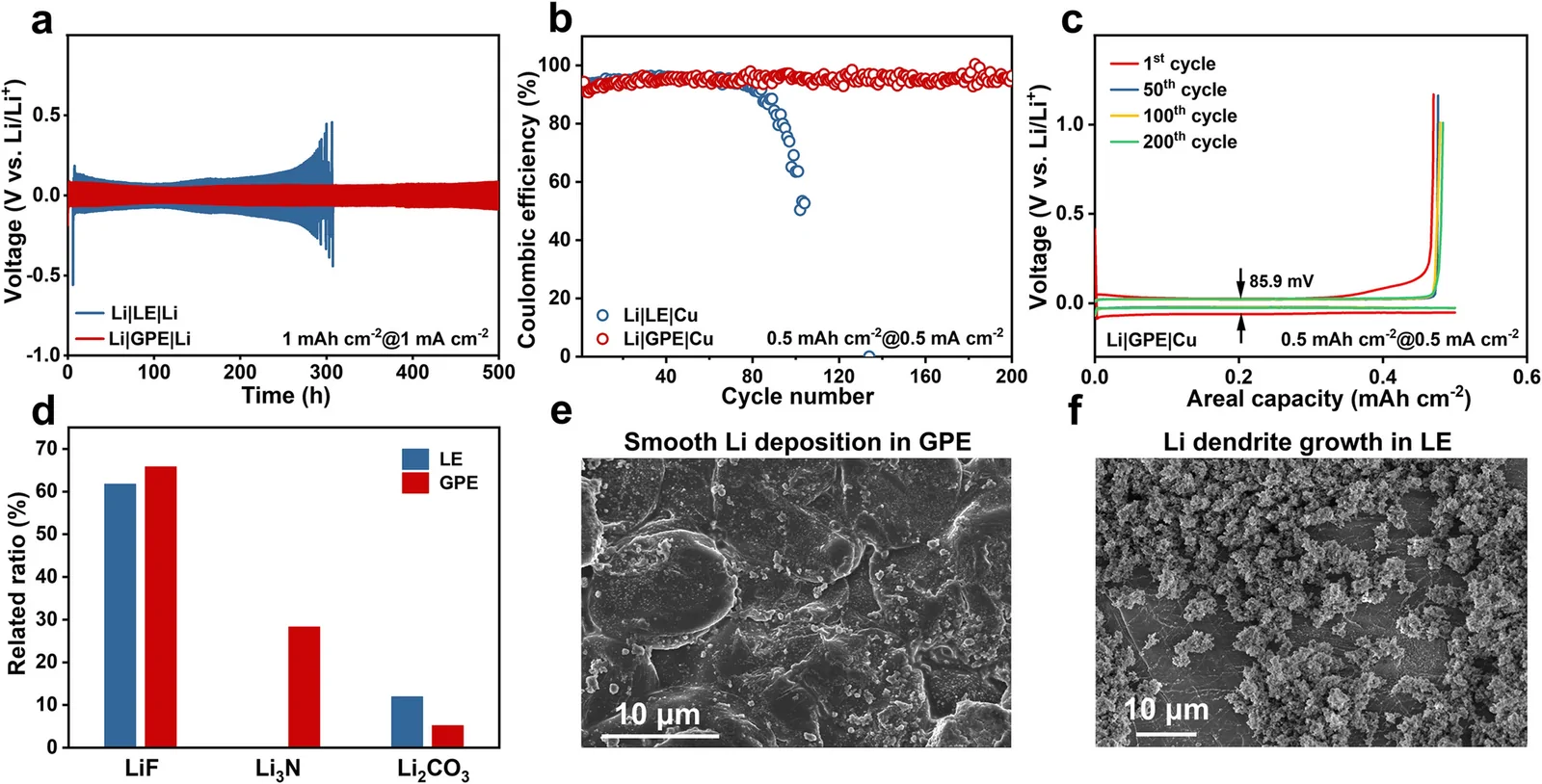
**a** Voltage–time profiles of symmetric Li|LE|Li and Li|GPE|Li cells with a cycling capacity of 1.0 mAh cm^−2^ at 1.0 mA cm^−2^. **b** Coulombic efficiency of asymmetric Li|LE|Cu and Li|GPE|Cu cells at 0.5 mA cm^−2^ with a capacity of 0.5 mAh cm^−2^. **c** Discharge–charge curves of asymmetric Li|GPE|Cu cell at 0.5 mA cm^−2^ with a capacity of 0.5 mAh cm^−2^. **d** A comparison of inorganic components in SEI formed in LE and GPE. SEM images showing the surface morphology of Li metal anodes after cycling in symmetric **e** Li|GPE|Li and **f** Li|LE|Li cells

The efficacy of GPE in regulating Li metal deposition is evaluated in symmetric cells, using Li metal as both working and counter electrodes (Li|GPE|Li). For comparison, symmetric cells using LE are also assembled (Li|LE|Li). The Li|GPE|Li cells maintain a low overpotential of 60 mV without short-circuiting for 500h at a current density of 1.0 mA cm^−2^ with a cycling capacity of 1.0 mAh cm^−2^ (Fig. 4a). In contrast, the Li|LE|Li cells show a rapid overpotential increase exceeding 100 mV and fail within 300 h under identical conditions. Asymmetric cells (Li|GPE|Cu) using Cu foil as the working electrode exhibit an initial CE of 94%, which stabilizes at 96.6% after 200 cycles at 0.5 mA cm^−2^ with a cycling capacity of 0.5 mAh cm^−2^ (Fig. 4b). Stable voltage profiles with minimal polarization reflect highly reversible Li plating/stripping in GPE (Fig. 4c), whereas Li|LE|Cu cells suffer severe CE fluctuation and rapid failure within 80 cycles due to uncontrolled and irreversible Li deposition. This improvement arises from the preferential reduction of LiNO_3_, whose lowest unoccupied molecular orbital (LUMO) energy level lies below that of LiPF_6_ and carbonate solvents [34, 35]. XPS analysis confirms the presence of Li_3_N (399.6 eV) along with NO_2_^−^ (403.7 eV) and NO_3_^−^ (407.3 eV) species in SEI on Li metal anode cycled in GPE, whereas these features are absent in LE-derived SEI (Figs. 4d and S16a). The C 1*s* and F 1*s* XPS spectra further reveal a higher fraction of inorganic LiF but reduced Li_2_CO_3_ content in GPE-derived SEI (Fig. S16b, c). The diminished Li_2_CO_3_ content indicates suppressed decomposition of carbonate solvents in GPE. Together, these findings demonstrate that LiNO_3_ promotes the formation of an inorganic-rich SEI, where high-modulus inorganic components act as electronic insulators but ionic conductors while reinforcing SEI integrity [36]. As a result, the GPE enables dense and dendrite-free Li deposition, in sharp contrast to the mossy, irregular dendrites observed with LE (Fig. 4e, f).

**Fig. 5:**
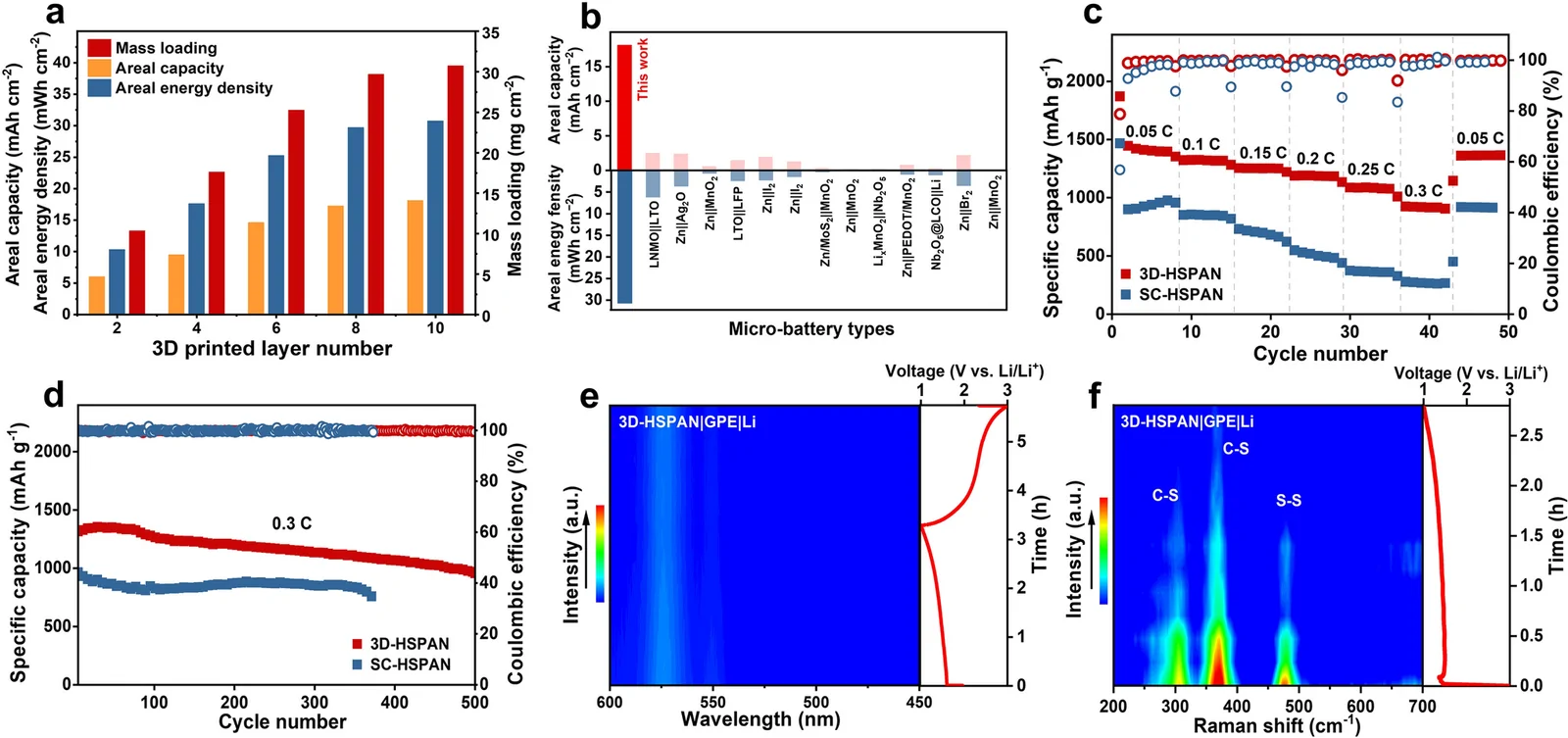
**a** The relationship of areal capacity and energy density with mass loading of the 3D-HSPAN cathodes at a current rate of 0.02 C in GPE. **b** A comparison of 3D-HSPAN|GPE|Li cells with reported planar microbatteries in areal capacity and areal energy density. **c** Rate performance of 3D-HSPAN|GPE|Li and SC-HSPAN|GPE|Li cells. **d** Cycling stability of 3D-HSPAN|GPE|Li and SC-HSPAN|GPE|Li cells at 0.3 Ce In situ UV–vis contour spectra of GPE in 3D-HSPAN|GPE|Li cells during cycling. **f** In situ Raman spectra of 3D-HSPAN cathode during the initial discharge process

Quasi-solid-state Li–S microbatteries are assembled by employing a 3D-HSPAN cathode against an ultra-thin Li metal anode (20 μm in thickness) in GPE (3D-HSPAN|GPE|Li). Using 3D-HSPAN cathode with an active mass loading of 8.33 mg cm^−2^, the 3D-HSPAN|GPE|Li cells deliver a discharge capacity of 1590 mAh g^−1^ (5.99 mAh cm^−2^) at a current rate of 0.02 C (1 C = 1675 mA g^−1^) (Fig. S17). Increasing the number of printed layers from four to ten results in an increment of mass loading from 16.05 to 37.1 mg cm^−2^ for the 3D-HSPAN cathode (Fig. 5a). This improvement yields high areal capacities up to 18.1 mAh cm^−2^ and areal energy density of 30.7 mWh cm^−2^, far surpassing most reported microbattery systems (Fig. 5b) [37–50]. Such performance highlights the great benefits of 3D printing in preserving electrode performance under critical mass loading conditions. For 3D-HSPAN|GPE|Li cells using 3D-HSPAN cathode with various mass loadings, they share a common feature of single-voltage plateaus in discharge–charge voltage curves, indicating a LiPS-free pathway of redox conversion (Fig. S17). This behavior is distinct from the dual-plateau pattern of conventional sulfur cathodes via a LiPS-intermediated solid–liquid–solid redox route [51].

Generally, the rate capability of conventional slurry-casting electrodes rapidly deteriorates with increasing mass loading and electrode thickness due to elevated diffusion limitations and polarization. For example, the SC-HSPAN cathode with a mass loading of 6.6 mg cm^−2^ delivers capacities of only 970–430 mAh g^−1^ at 0.05–0.2 C and fails at higher rates of 0.25–0.3 C in quasi-solid-state cells (SC-HSPAN|GPE|Li) (Fig. 5c), whereas the 3D-HSPAN|GPE|Li cells using 3D-HSPAN cathode, even with a higher mass loading of 8.9 mg cm^−2^, achieve 1.4–3.5 times greater capacity retention, retaining 1400–915 mAh g^−1^ across 0.05–0.3 C due to enhanced electrode kinetics (Fig. 5c). When cycled at 0.3 C, this cell retains a high capacity of 1280 mAh g^−1^ with 75% capacity retention and nearly 100% CE after 500 cycles, in contrast to the rapid failure of the SC-HSPAN|GPE|Li cell with much lower capacities (Fig. 5d). After one month of storage, the 3D-HSPAN|GPE|Li cells exhibit negligible variation in interfacial resistance and minimal capacity decay, indicating robust interfacial compatibility between the electrolyte and the electrodes over extended periods (Fig. S18). Moreover, the cells exhibit stable retention of open-circuit voltage (OCV) and negligible capacity decay at 30–60 °C over 7 days of standing, demonstrating good tolerance to self-discharge (Fig. S19).

Cyclic voltammetry (CV) analysis validates a direct solid–solid redox conversion mechanism for the 3D-HSPAN cathode (Fig. S20). Two cathodic peaks appear at *ca.* 2.0 and 1.7 V, corresponding to the stepwise reduction of short-chain S to Li_2_S_2_ and Li_2_S, while an anodic peak near *ca.* 2.4 V reflects the oxidation of Li_2_S back to S bonding to PAN chains [52, 53]. The absence of characteristic LiPS peaks indicates a redox pathway free from soluble intermediates. This mechanism is further supported by featureless in situ UV–vis spectra of 3D-HSPAN throughout the discharge–charge cycle in GPE (Fig. 5e) [51, 54]. The in situ Raman spectroscopy reveals the gradual disappearance of vibrational bands associated with C–S in-plane bending (308 cm^−1^), C–S deformation (370 cm^−1^), and S–S bond (464 cm^−1^), without detectable LiPS signals during discharge in GPE (Fig. 5f) [8, 25, 55]. The elimination of LiPS reduces performance deterioration caused by rapid electrolyte saturation, LiPS shuttle effect, and degradation of Li metal anode, all of which are critical for the efficient operation of Li–S microbatteries. Furthermore, the absence of LiPS prevents nucleophilic attack on carbonate solvents, ensuring the 3D-HSPAN cathode is compatible with widely used carbonate-based electrolytes [54].

### Plasmonic Enhancement of Li–S Redox Kinetics in 3D-HSPAN|GPE|Li Cells

**Fig. 6:**
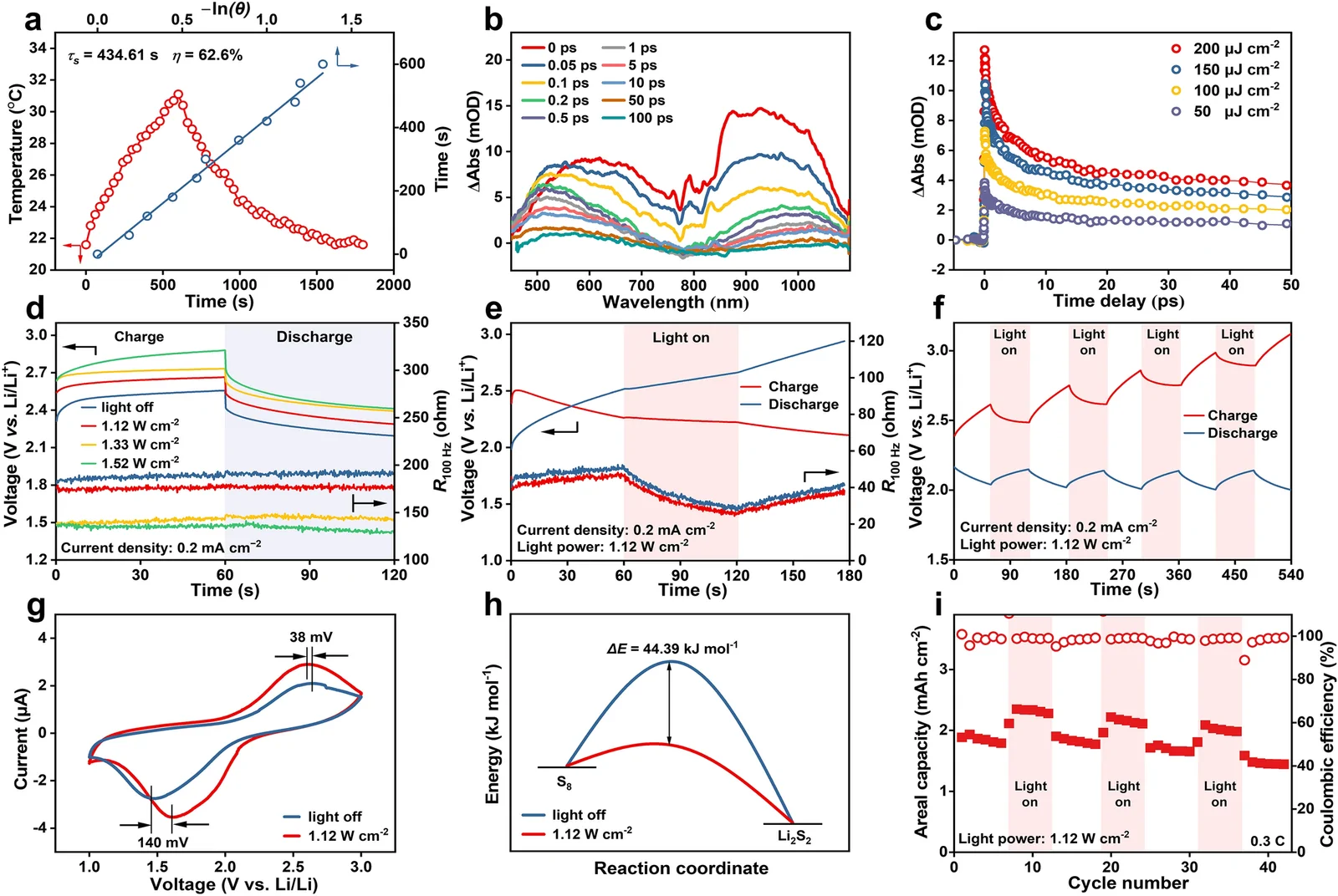
**a** Photothermal response of MXene aqueous colloid under NIR irradiation at *λ* = 808 nm for various durations. The heat transfer time constant (*τ*_*s*_) is obtained from the linear fitting of the cooling curve. **b** Time-resolved vis–NIR transient absorption spectra of MXene at different delay times with a pump beam at *λ* = 800 nm. **c** Kinetic traces of the excited-state absorption decay of MXene. **d** The impedance of the 3D-HSPAN electrode at a fixed frequency of 100 Hz during charge–discharge at different NIR light powers. **e** NIR light on/off response of the impedance of the 3D-HSPAN electrode at 100 Hz during the charge–discharge process. **f** NIR light on/off response of cell voltage during the charge–discharge process. **g** CV curves of 3D-HSPAN|GPE|Li cells under NIR irradiation and dark conditions. **h** The relative activation energies for the S_8_-to-Li_2_S_2_ conversion. **i** NIR light on/off response of capacity retention and coulombic efficiency of 3D-HSPAN|GPE|Li cells

The 3D-HSPAN cathode features a highly open architecture integrated with MXene, an efficient plasmonic material that combines high conductivity with pronounced localized surface plasmon resonance (LSPR) across a broad spectrum [56, 57]. This design offers the opportunity to improve the electrochemical performance of 3D-printed electrodes under external electromagnetic irradiation. Using Ti_3_C_2_T_*x*_ MXene as a proof-of-concept, it exhibits strong near-infrared (NIR) absorption with a broad and intense plasmonic resonance centered at *λ* = 780 nm (Fig. S21a), exhibiting a superior extinction coefficient (*ε* = 26.44 L g^−1^ cm^−1^) to conventional plasmonic materials such as Au nanoparticles (*ε* = 13.1 L g^−1^ cm^−1^) (Fig. S21b, c) [58]. This MXene achieves a high photothermal conversion efficiency (*η*) of *ca.* 62.6% under NIR irradiation at 2.57 W cm^−2^ and *λ* = 808 nm (Fig. 6a). Beyond its significant photothermal effect, femtosecond transient absorption spectroscopy (fs-TAS) further suggests ultra-fast generation of hot carriers within 250 fs upon excitation at *λ* = 800 nm, followed by decay over *ca.* 10 ps via electron–phonon scattering (Fig. 6b, c). This carrier lifetime exceeds that of traditional plasmonic metals and may be further prolonged under a directional electric field, potentially enhancing hot-carrier participation in electrochemical reactions [59]. These benefits offer the potential of utilizing LSPR-driven photothermal and hot-carrier effects to boost the redox performance of the 3D-HSPAN cathode.

The positive impact of LSPR on the redox kinetics of the 3D-HSPAN cathode is probed by EIS measurement. At a fixed frequency of 100 Hz, the impedance is predominantly governed by interfacial *R*_ct_. Increasing NIR power induces a pronounced decrease in *R*_ct_, indicating accelerated charge-transfer kinetics (Fig. 6d). The rapid and reversible reduction of *R*_ct_, coupled with suppressed voltage polarization during NIR light on–off cycles, demonstrates the beneficial role of plasmonic MXene in accelerating interfacial charge transfer and electrode kinetics (Fig. 6e, f). CV analysis further validates the beneficial effect of NIR irradiation on the redox activity of 3D-HSPAN, evidenced by reduced separation between cathodic and anodic peaks and increased redox currents compared to NIR-off conditions (Fig. 6g). Under NIR irradiation at 1.12 W cm^−2^, the activation energy (*E*ₐ) decreases by 44.39  kJ mol^−1^ for sulfur reduction and 3.73  kJ mol^−1^ for the reverse oxidation (Fig. 6h, Fig. S22). This phenomenon suggests that MXene enhances the redox activity of 3D-HSPAN via a synergistic effect of LSPR-induced photothermal heating and hot-carrier injection, as thermal effects alone cannot alter *E*ₐ. Consequently, NIR irradiation at 1.12 W cm^−2^ and *λ* = 808 nm reduces internal resistance by *ca.* 67% and lowers voltage polarization from 0.59 to 0.45 V, yielding over 23% capacity improvements for the 3D-HSPAN electrode with 5 wt.% MXene at 0.3 C at room temperature (Figs. 6i and S23). When the MXene content decreases to 1–2.5 wt%, the capacity enhancement ratio upon NIR illumination reduces to 4.6%–11.3% (Fig. S24a–c). Increasing the MXene loading beyond 5 wt% (*e.g.*, 7.5 and 10 wt%) still yields noticeable capacity enhancement, but the gain declines to 14.4%–17.5%. This reduction arises from excessive MXene loading, which induces overheating under NIR irradiation to deteriorate electrolyte stability (Fig. S24d, e). A similar trend is observed with increasing NIR intensity or 3D-HSPAN electrode with 5 wt% MXene. As the NIR intensity increases from 0.78 to 1.12 W cm⁻^2^, the capacity enhancement ratio rises from 6.5% to 20.6% (Fig. S25). When the irradiation power exceeds 1.33 W cm⁻^2^, the capacity enhancement remains evident but slightly decreases to 19.3%. This decline originates from overheating at excessive NIR power, which compromises electrolyte stability and accelerates parasitic side reactions. After cycling, the Ti 2*p* XPS spectra of the 3D-HSPAN electrode retain their original features, without noticeable increase in Ti–O peak intensity, ruling out MXene oxidation during cycling. The observed Ti–S bonds arise from chemical interactions between MXene and the discharge product (Li_2_S) (Fig. S26).

### Assembly and Performance of Transparent Quasi-Solid-State Microbatteries

**Fig. 7:**
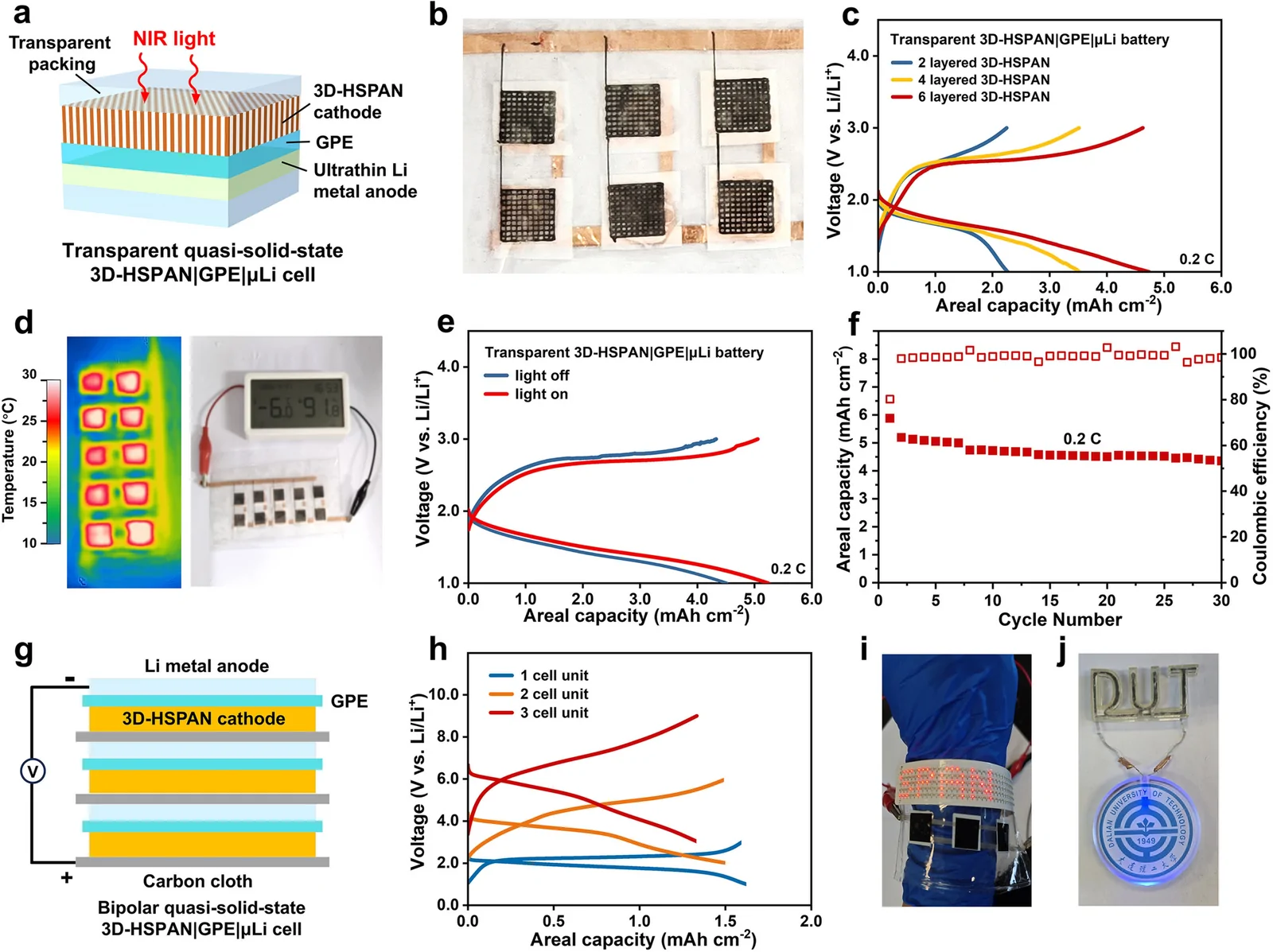
**a** Schematic illustration and **b** optical image of transparent quasi-solid-state 3D-HSPAN|GPE|μLi microbatteries. **c** Discharge–charge voltage curves of 3D-HSPAN|GPE|μLi cells using 3D-HSPAN cathodes with varying printed layers and mass loading at 0.2 C. **d** Infrared thermography and optical image of transparent 3D-HSPAN|GPE|μLi cells under simulated solar illumination, powering an LCD at − 6 °C. **e** Discharge–charge voltage curves of 3D-HSPAN|GPE|μLi microbatteries under solar illumination and in the dark at 0.2 C. **f** Cycling performance of transparent 3D-HSPAN|GPE|μLi microbatteries at 0.2 C. **g** Schematic diagrams and **h** Discharge–charge voltage curves of bipolar 3D-HSPAN|GPE|μLi microbatteries composed of two and three stacked cell units at 0.8 mA cm^−2^. Optical images of **i** flexible and **j** shape-customized 3D-HSPAN|GPE|μLi microbatteries designed for powering electronics

Transparent quasi-solid-state Li–S microbatteries are demonstrated by assembling a grid-patterned 3D-HSPAN cathode with an ultra-thin Li metal anode (20 μm in thickness) in GPE, followed by encapsulating within transparent PET films (Fig. 7a, b). Such cells deliver an areal capacity of 2.3–4.8 mAh cm^−2^ at 0.2 C, as the printed layer number of the 3D-HSPAN cathode increases from two to six layers (Fig. 7c). Benefiting from the photothermal effect of MXene across a broad solar spectrum, the transparent quasi-solid-state 3D-HSPAN|GPE|μLi microbatteries exhibit a temperature rise of 15 °C under one-sun illumination. This enables cell operation at sub-zero temperature, holding promise in cold outdoor IoT applications (Fig. 7d). Simultaneously, the areal capacities of such cells are increased by 12% under similar illumination conditions at 0.2 C, with stable operation for 30 cycles (Fig. 7e, f). The output voltage and areal energy density of these microbatteries can also be readily scaled by internally stacking individual cell units to form a bipolar battery configuration (Fig. 7g). The bipolar cells allow for raising the average cell voltages to 3.8 and 5.9 V by stacking two and three cell units, respectively. This design may overcome the limitation of Li–S cells in output voltage (Fig. 7h). The transparent quasi-solid-state 3D-HSPAN|GPE|μLi microbatteries show excellent flexibility with high-capacity retention (1130–1360 mAh g^−1^) upon deformation from flat state to 180° bending, holding promise in wearable and portable electronics (Figs. 7i and S27). Shape-customized quasi-solid-state microbatteries are also demonstrated by a DUT-shaped transparent cell for powering a light display (Fig. 7j).

## Conclusion

In summary, we demonstrated the design of a high-energy quasi-solid-state Li–S microbattery by integrating additive manufacturing with high-energy sulfur redox chemistry. Direct ink writing produces HSPAN cathodes with a highly ordered and hierarchically open porous framework. This improvement enables ultra-high sulfur loadings up to 37.1 mg cm^−2^ while maintaining efficient ionic and charge transport in thick electrodes. The incorporation of plasmonic MXene generates a significant photothermal effect and hot-carrier injection via the LSPR effect under NIR irradiation. It reduces internal resistance by *ca.* 67% and lowers the activation energy of Li–S redox conversion in the absence of LiPS intermediates, yielding over 23% capacity improvements. On this basis, a LiNO_3_ sustained-release carbonate-based gel polymer electrolyte is also developed to ensure Li dendrite suppression. The resultant quasi-solid-state 3D-HSPAN|GPE|Li microbatteries deliver high areal capacities over 18.1 mAh cm^–2^ and exceptional areal energy densities up to 30.7 mWh cm^−2^. The cells exhibit stable cycling over 500 cycles with 75% capacity retention, with nearly 100% Coulombic efficiency and greatly improved rate capability. Moreover, transparent, flexible, and shape-customizable microbatteries are also assembled, demonstrating good adaptability in wearable electronics and low-temperature applications. This work establishes a system-level engineering platform for advancing microenergy storage solutions through synergistic integration of additive manufacturing, high-energy redox chemistry, and light-harvesting physics.

## Supplementary Information

Below is the link to the electronic supplementary material.

Supplementary file1 (DOCX 4284 KB)
