# Supplementary material for: 3D Printing Plasmonic-Enhanced Sulfurized Polyacrylonitrile Cathodes for High-Energy Li–S Microbatteries
Source: Nanomicro Lett. 2026 May 4;18:353. doi: 10.1007/s40820-026-02204-w (PMC13139552; doi:10.1007/s40820-026-02204-w)
Supplement: Supplementary file 1 — Supplementary file1 (DOCX 4284 KB) [file 40820_2026_2204_MOESM1_ESM.docx]

Supporting Information for

**3D Printing Plasmonic-Enhanced Sulfurized Polyacrylonitrile Cathodes for High-energy Li–S Microbatteries**

Yu Liu^1^, Penghao Fu^1^, Jieshan Qiu^3^, and Zhiyu Wang^1, 2,^ *

^1^ State Key Lab of Fine Chemicals, Frontiers Science Center for Smart Materials Oriented Chemical Engineering, Liaoning Key Lab for Energy Materials and Chemical Engineering, School of Chemical Engineering, Dalian University of Technology, Dalian 116024, P. R. China

^2^ School of Chemical Engineering and Technology, Xinjiang University, Urumqi 830046, P. R. China

^3^ College of Chemical Engineering, Beijing University of Chemical Technology, Beijing 100029, P. R. China

*Corresponding author. E-mail: [zywang@dlut.edu.cn](mailto:zywang@dlut.edu.cn) (Zhiyu Wang)

**S1 Experimental Section**

**Synthesis of Ti_3_C_2_T*_x_* MXene**

The Ti_3_C_2_T*_x_* MXene was synthesized by etching 5.0 g of Ti_3_AlC_2_ MAX phase in a mixed solution of LiF (13.2 g) and hydrochloric acid (6 M, 200 mL) for 72 h at 45 °C. The obtained products were harvested by several centrifugation-rinsing cycles with deionized (DI) water, followed by ultrasonic treatment in DI water for 1 h [S1].

**Material characterization**

The morphology of the samples was examined by scanning electron microscopy (SEM, JSM-7900). The texture and composition of the materials were characterized by X-ray diffraction (XRD, Rigaku D/MAX-2400, Cu Kα), X-ray photoelectron spectroscopy (XPS, Shimadzu AXIS SUPRA+), Raman spectroscopy (Horiba LabRAM), Fourier transform infrared spectroscopy (FTIR, Thermo Fisher iS50), and an elemental analyzer (Elementar Vario EL cube). The rheological property of the ink was measured by a rheometer (DHR2, TA Instruments-Waters LLC). The localized surface plasmon resonance (LSPR) properties of MXene were measured by UV-vis spectroscopy (PerkinElmer Lambda 750) and femtosecond transient absorption spectroscopy (fs-TAS).

**Measurement of ionic conductivity**

The ionic conductivities (*σ*) of GPE and LE were obtained from the impedance spectroscopy of the symmetric cells employing stainless-steel (SS) foils as the working electrodes by the following equation:

*σ* = *L* / (*A* × *R*) (S1)

where *R* is the ohmic resistance, *A* is the electrode area, and *L* is the space between two SS electrodes. The data points from –20 °C to 60 °C were collected on a CHI 760E electrochemical workstation in a frequency range of 100 kHz–0.01 Hz. The *E*_a_ was calculated by the Arrhenius law:

*σ* = *B* exp (−*E*_a_ / *RT*) (S2)

where *B* is the frequency factor, *R* is the molar gas constant, and *T* is the absolute temperature.

**Measurement of Li^+^ transference number**

The Li^+^ transference numbers (*t*_Li⁺_) of GPE and LE were determined by the potentiostatic polarization technique using Li||Li symmetric cells on a Vertex C.EIS electrochemical workstation (IVIUM). The *t*_Li⁺_ was calculated by the following equation:

*t*_Li⁺_ = [*I*_ss_ × (∆V – *I*_0_ × *R*_0_)] / [*I*_0_ × (∆V – *I*_ss_ × *R*_ss_)] (S3)

where ΔV is the applied bias voltage, while the initial current *(I*_0_) and the steady current (*I*_ss_) can be obtained under a direct current voltage of 10 mV. The interface resistance before (*R*_0_) and after polarization (*R*_ss_) was determined through AC impedance with an amplitude of 10 mV in the frequency range of 100 kHz–0.01 Hz.

**Measurement of electrochemical stability window and Tafel plots**

The electrochemical stability window (ESW) of the GPE and LE was measured by linear sweep voltammetry (LSV) scan of an asymmetric coin cell utilizing Li metal foil against SS at a scan rate of 5 mV s^−1^. The Li||Li symmetric cells were used to get Tafel plots within −0.1–0.1 V at a scan rate of 0.1 mV s^−1^.

***In-situ* measurement and analysis**

*In-situ* Raman and UV-vis analysis were performed using 3D-HSPAN as the working electrode against Li metal in GPE. For comparison, *In-situ* tests were also conducted for the SC-HSPAN cathodes against the Li metal anode in GPE. *In-situ* UV-vis analyses were conducted by using a customized battery device with a quartz window in reflection mode on a UV-vis-NIR spectrometer (PerkinElmer Lambda 750). The Li metal with a circular hole was used against the working electrode to enable access of the electrolyte to UV light. The UV-vis spectra were recorded every 300 s in a wavelength range of 450–600 nm during cell operation at a potential range of 1.0–3.0 V at 0.2 C on a LAND CT2001A battery tester. The intensity of the UV-vis signal was positively correlated with LiPS concentration based on the Beer-Lambert law. *In-situ* Raman analysis was performed by using a 3D-HSPAN cathode against Li metal foil in GPE in a homemade device with a quartz window on a Raman spectrometer (Zhongke Kaili, China) with a laser wavelength of 532 nm every 10 min. Meanwhile, the cell was discharged at 0.2 C on a LAND CT2001A battery tester. *In-situ* EIS measurements were conducted by using a 3D-HSPAN cathode against Li metal foil in GPE on a CHI 760E electrochemical workstation. The EIS patterns were recorded at different voltages in a frequency range of 100 kHz–0.01 Hz with 10 mV amplitude. Distribution of relaxation times (DRT) and the distribution of diffusion times (DDT) results were derived from these impedance data by using DRT tools based on Bayesian regularization with continuous function discretization.

**Calculation of extinction coefficient and photothermal conversion efficiency of MXene [S2]**

The extinction coefficient (*ε*) was calculated by performing a linear fit of UV-vis absorption spectra of MXene aqueous solution with varying concentrations at a wavelength (*λ*) of 780 nm based on the Beer–Lambert law:

$A\left( \lambda\right)=\varepsilon CL$ (S4)

where *A* is the absorbance at a certain *λ*, *L* is the optical path length, and *C* is the concentration of MXene aqueous solution.

The photothermal conversion efficiency (*η*) was calculated as the ratio of the energy converted from light to heat to the total received optical energy. The energy balance of the entire system adheres to the following equation:

$\sum_{i}m_{i}C_{p,i}\frac{dT}{dt}=Q_{MXene}+Q_{Dis}-Q_{surr}$ (S5)

where *m* and *C_p_* are the mass and heat capacity of water, *T* is the temperature of MXene aqueous solutions, *Q*_MXene_ is the optical energy captured by MXene, *Q*_Dis_ is the baseline energy of the sample cell, and *Q*_surr_ is the heat energy dissipated from the system to the surrounding environment. *Q*_MXene_, *Q*_Dis,_ and *Q*_surr_ are determined by the following equations:

$Q_{MXene}=I\left( 1-{10}^{-A_{\lambda}} \right)\eta$ (S6)

$Q_{Dis}=hS(T_{max,water}-T_{surr,water})$ (S7)

$Q_{Surr}=hS(T-T_{surr})$ (S8)

where *I* is the laser power density, *A*_λ_ is the absorbance of MXene at *λ* = 808 nm, *η* is the photothermal conversion efficiency of MXene, *T* is the temperature of the system surface, *T*_surr_ is the temperature of the surrounding environment, *T*_max, water_ and *T*_surr, water_ are the temperature of pure water after exposure to the NIR light and under ambient conditions, respectively. The *h* is the heat transfer coefficient, and *S* is the surface area of the heat transfer.

The driving force for heat exchange (*θ*) and time constant of the sample system (*τ*_s_) were calculated by the following equations:

$\theta=\frac{T-T_{surr}}{T_{max}-T_{surr}}$ (S9)

$\tau_{s}=\frac{\sum_{i}m_{i}C_{p,i}}{hS}$ (S10)

During the cooling period of the MXene aqueous solution, no additional energy is input, namely:

$Q_{MXene}+Q_{Dis}=hS\left( T_{max}-T_{surr} \right)$ (S11)

The heat transfer time constant can be determined from the cooling curve, and integrating gives the expression as follows:

$dt=-\tau_{s}\frac{d\theta}{\theta}$ (S12)

$t=-\tau_{s}\ln\theta$ (S13)

On this basis, the *η* was determined by the following equation:

$\eta=\frac{hS\left( T_{max}-T_{surr} \right)-Q_{Dis}}{I(1-{10}^{-A_{\lambda}})}$ (S14)

**S2 Supplementary Figures**


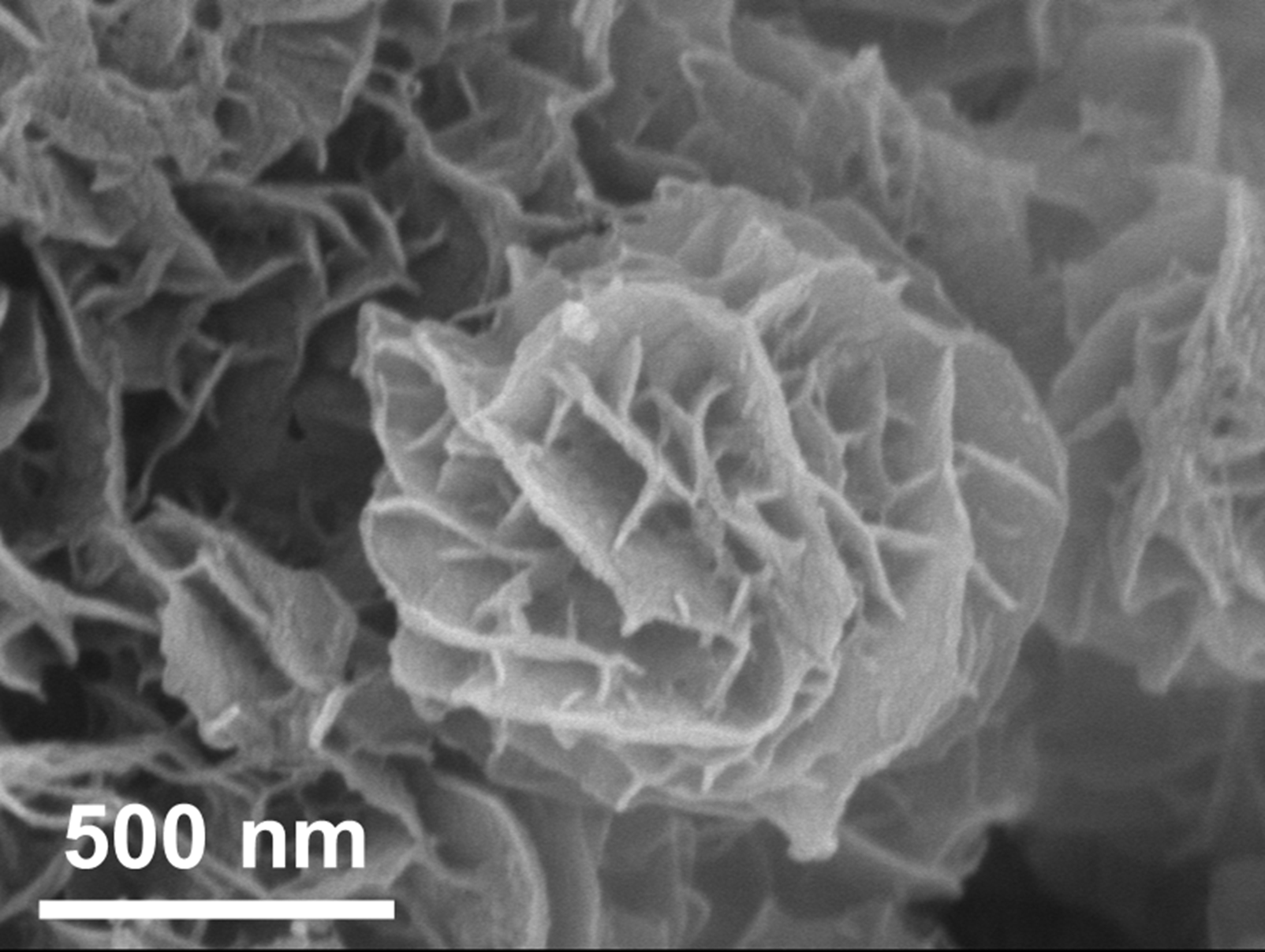


**Fig. S1** SEM image of HPAN


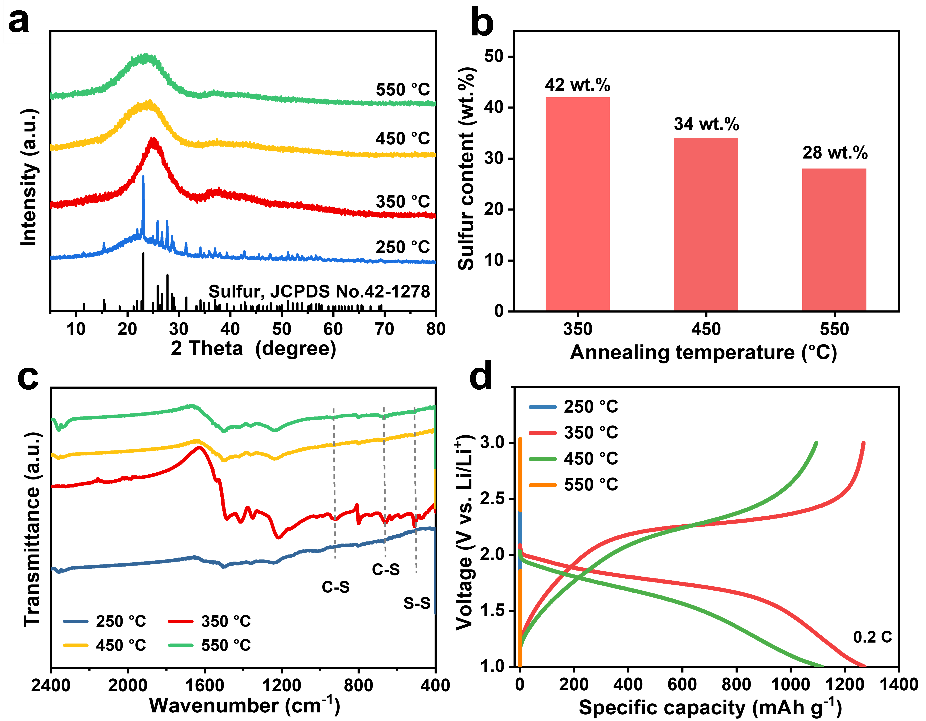


**Fig. S2 a** XRD patterns, **b** sulfur content, **c** FT-IR spectra, and **d** discharge-charge voltage curves of HSPAN cathodes synthesized at 250, 350, 450, and 550 ^o^C

**
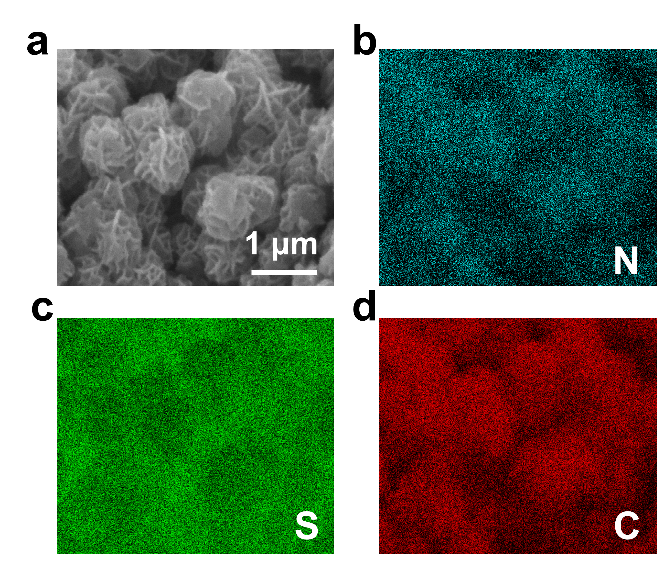
**

**Fig. S3 a** SEM image and **b, c, d** corresponding element mapping of HSPAN

**
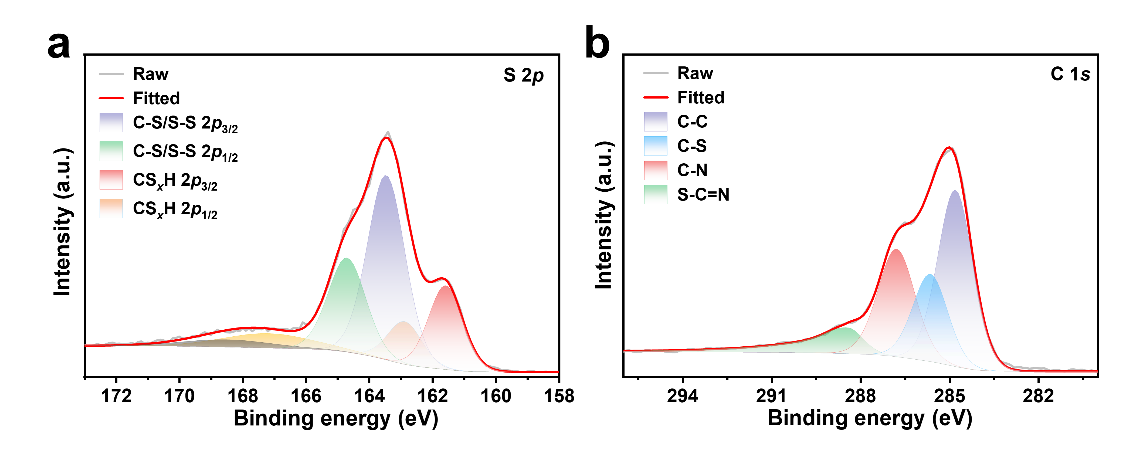
**

**Fig. S4 a** S 2*p* and **b** C 1*s* XPS spectra of HSPAN

**
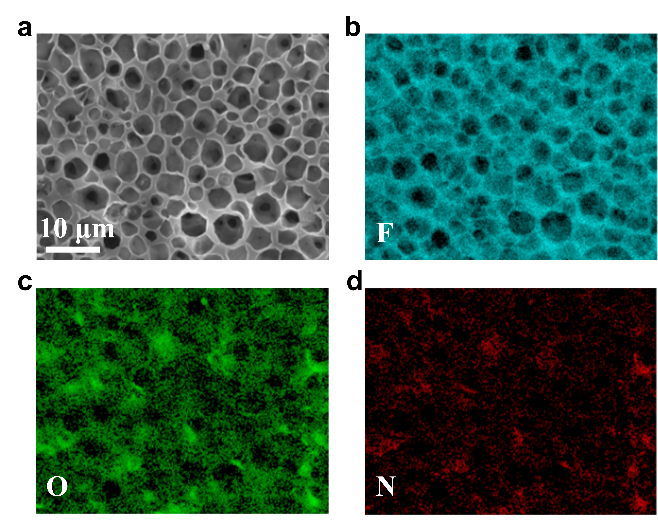
**

**Fig. S5 a** SEM image and **b, c, d** corresponding element mapping of LiNO_3_@PVDF-HFP membrane

**
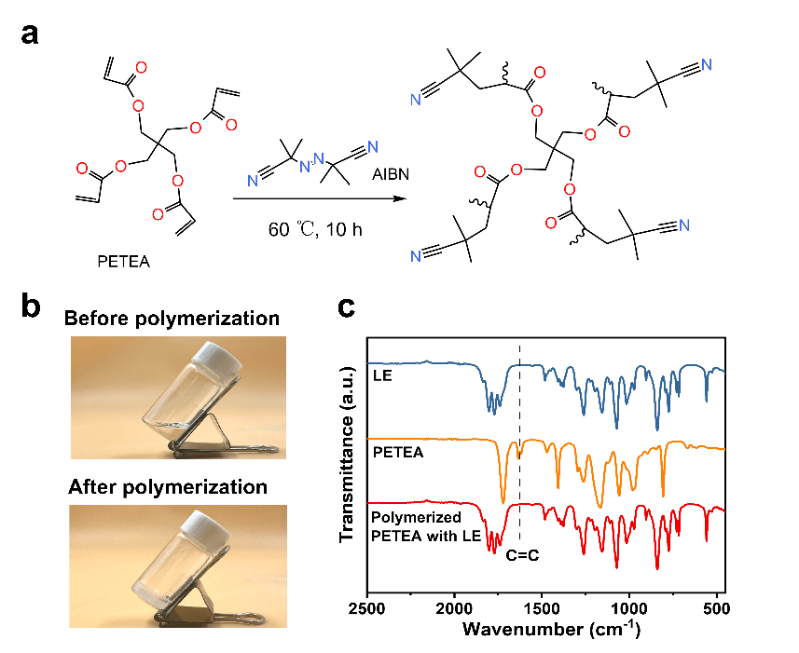
**

**Fig. S6 a** Thermal-driven radical polymerization route of PETEA with AIBN as a radical initiator. **b** Optical images of LE containing PETEA and AIBN before and after thermal polymerization. **c** FTIR spectra of LE, PETEA, and polymerized PETEA with LE

**
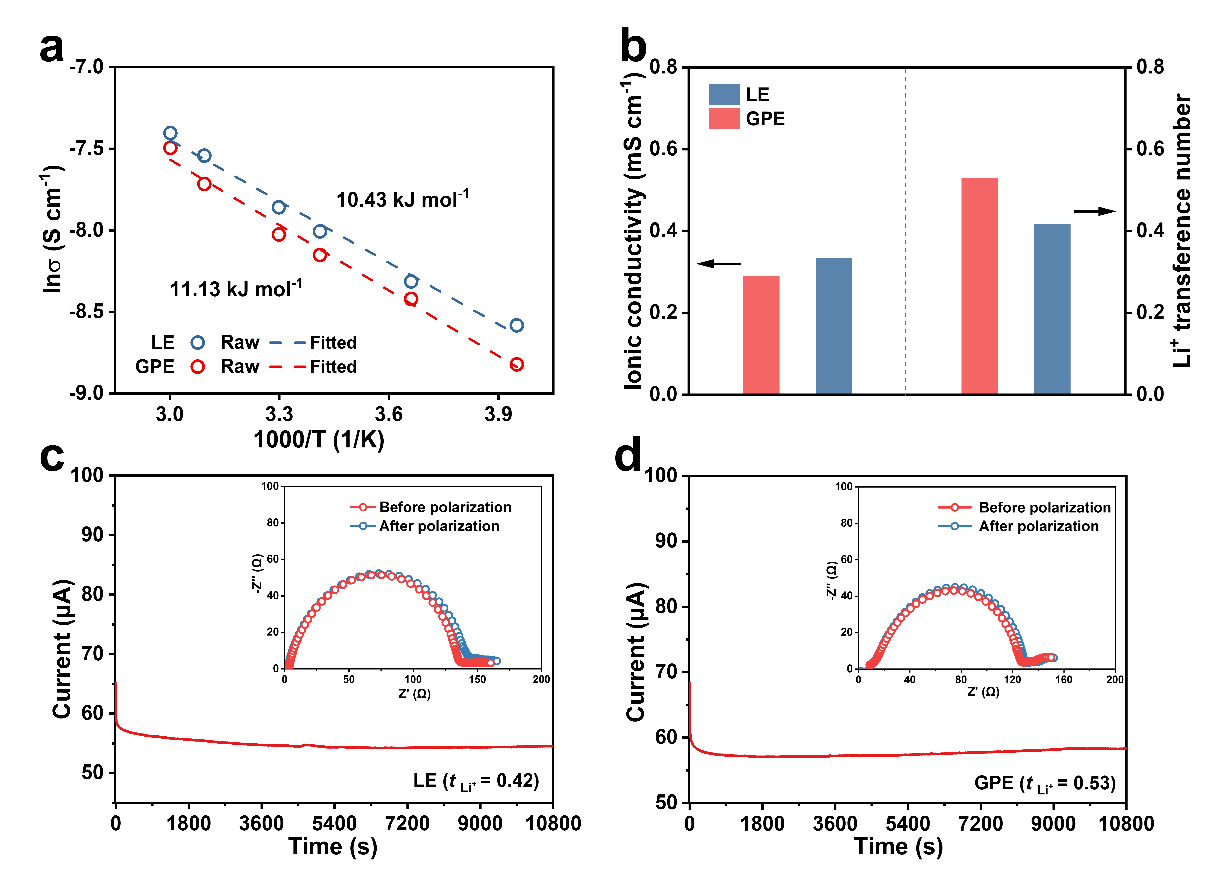
**

**Fig. S7 a** Temperature-dependent ionic conductivity of GPE and LE, fitted using the Arrhenius transport model. **b** Comparison of GPE and LE in ionic conductivity and Li^+^ transference number. The chronoamperometry profile of **c** Li|LE|Li and **d** Li|GPE|Li cells under a polarization voltage of 10 mV. The insets are the corresponding EIS spectra before and after polarization in a frequency range of 100 kHz–0.01 Hz

**
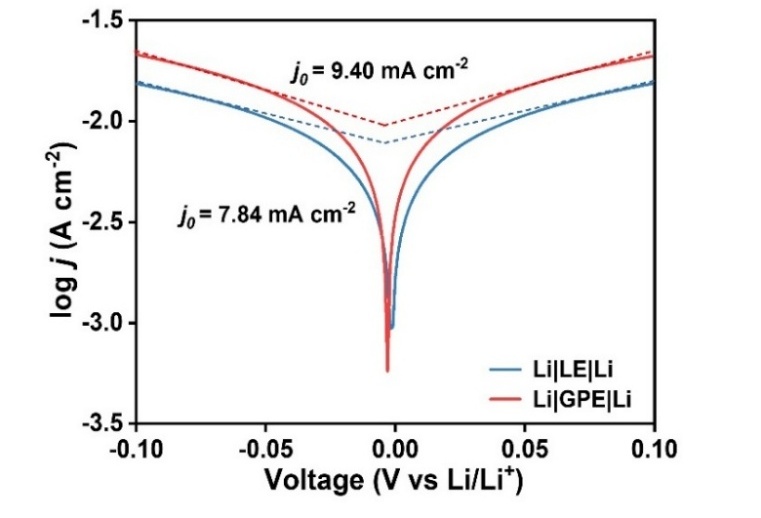
**

**Fig. S8** Exchange current density of Li|LE|Li and Li|GPE|Li cells at a scan rate of 0.1 mV s^−1^

**
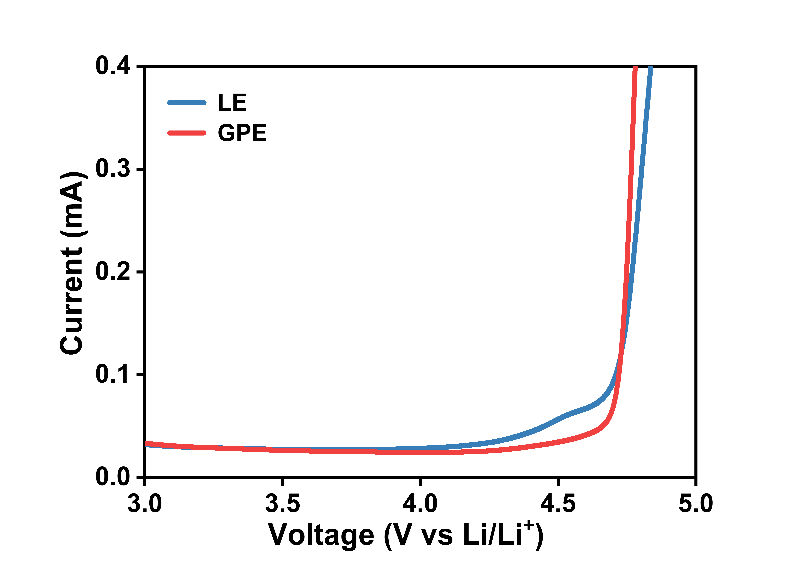
**

**Fig. S9** LSV curves of LE and GPE measured in asymmetric Li||SS cells at a scan rate of 5 mV s^−1^

**
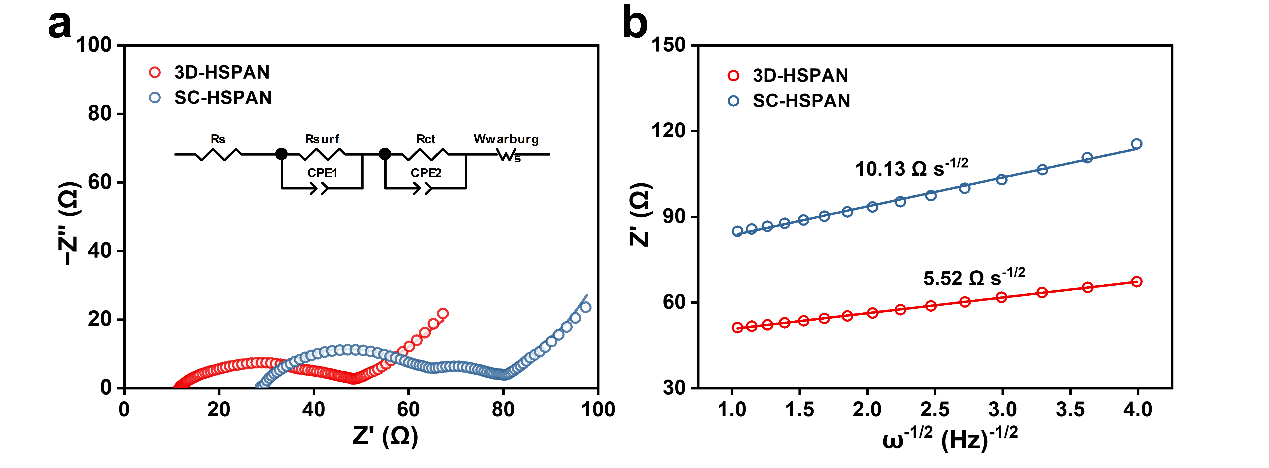
**

**Fig. S10** **a** EIS patterns of 3D-HSPAN and SC-HSPAN electrodes after discharge in a frequency range of 100 kHz–0.01 Hz. **b** The correlation between *Z*’ and *ω^–^*^1/2^ at low frequency derived from EIS patterns

**
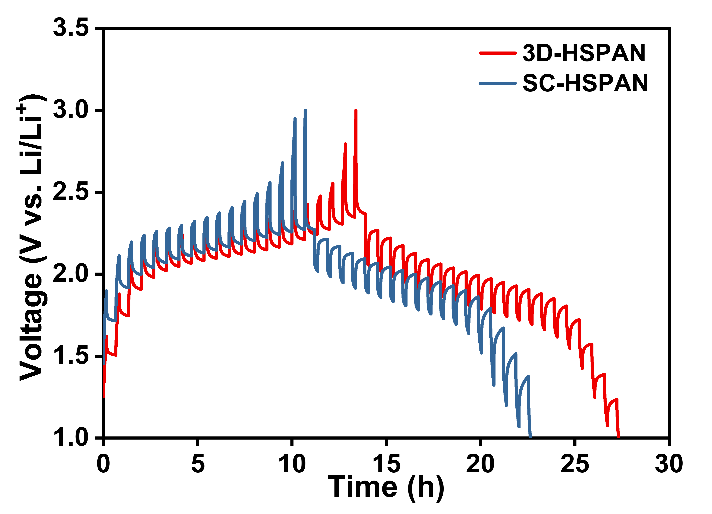
**

**Fig. S11** GITT curves of 3D-HSPAN and SC-HSPAN cathodes in GPE

**
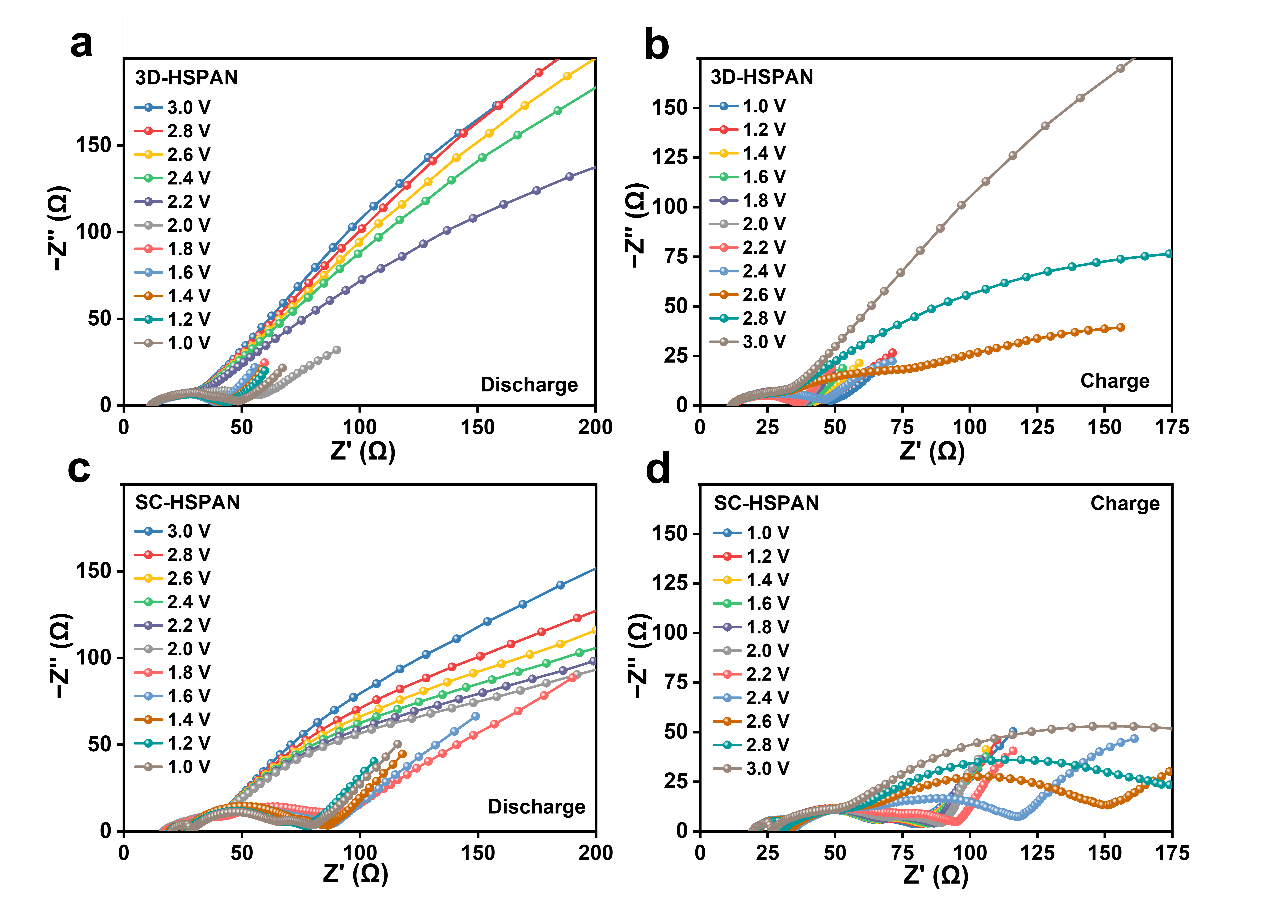
**

**Fig. S12** *In-situ* EIS spectra of **a, b** 3D-HSPAN and **c, d** SC-HSPAN cathodes at different states of discharge and charge in a frequency range of 100 kHz–0.01 Hz


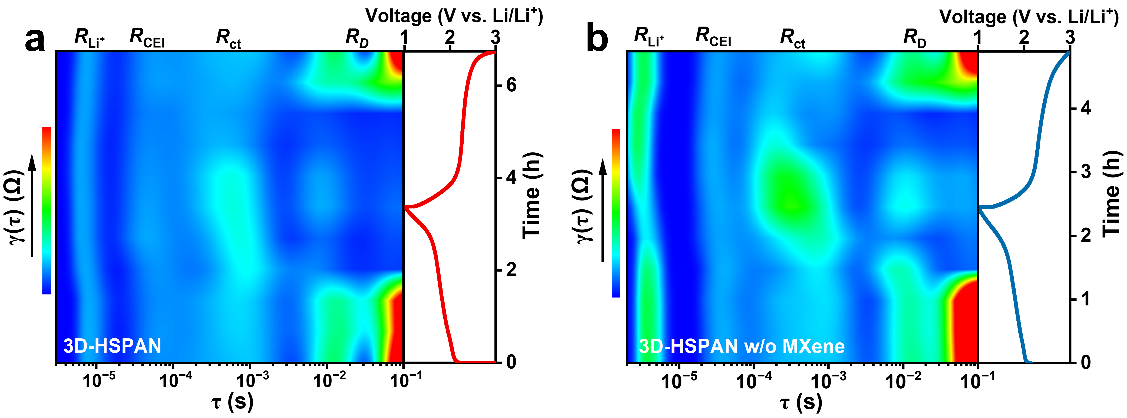


**Fig. S13** DRT profiles derived from *in-situ* EIS patterns of 3D-HSPAN **a** with and **b** without MXene in GPE


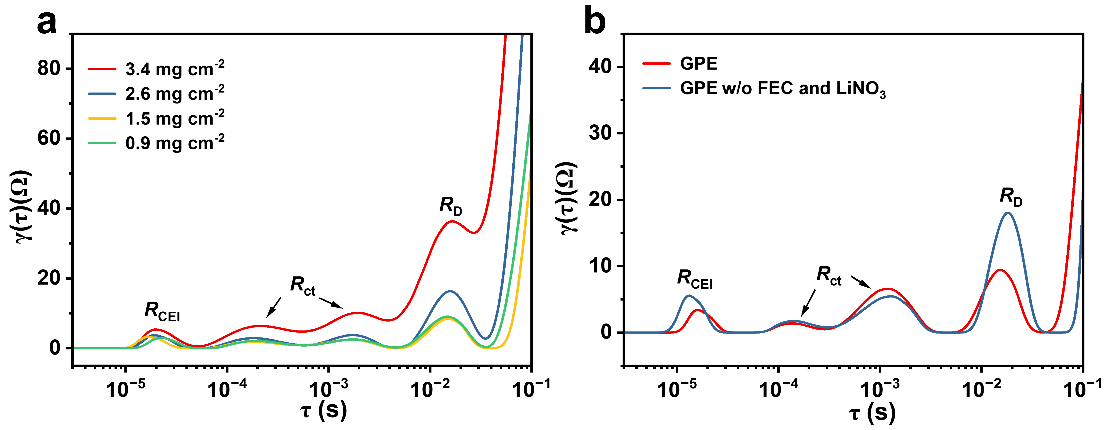


**Fig. S14** DRT curves derived from EIS patterns of 3D-HSPAN electrodes **a** with varying mass loadings in GPE and **b** in GPE with and without FEC/LiNO_3_ additives


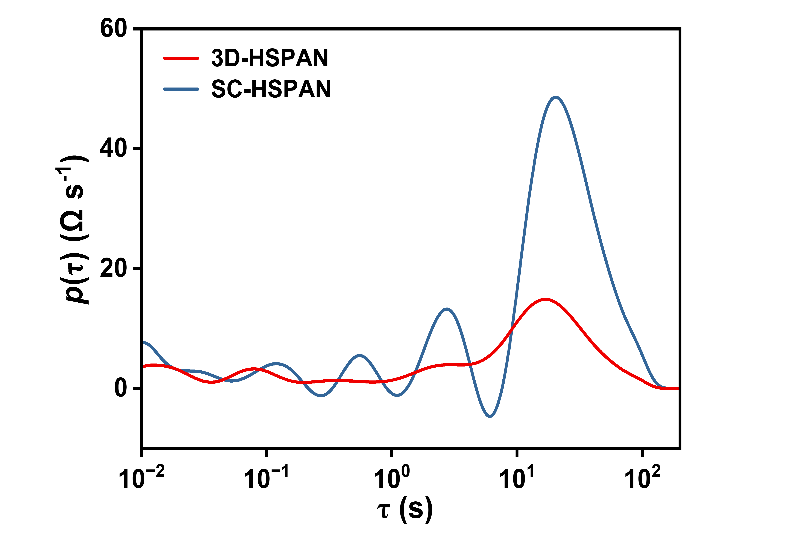


**Fig. S15** DDT curves derived from EIS patterns of 3D-HSPAN and SC-HSPAN cathodes

**
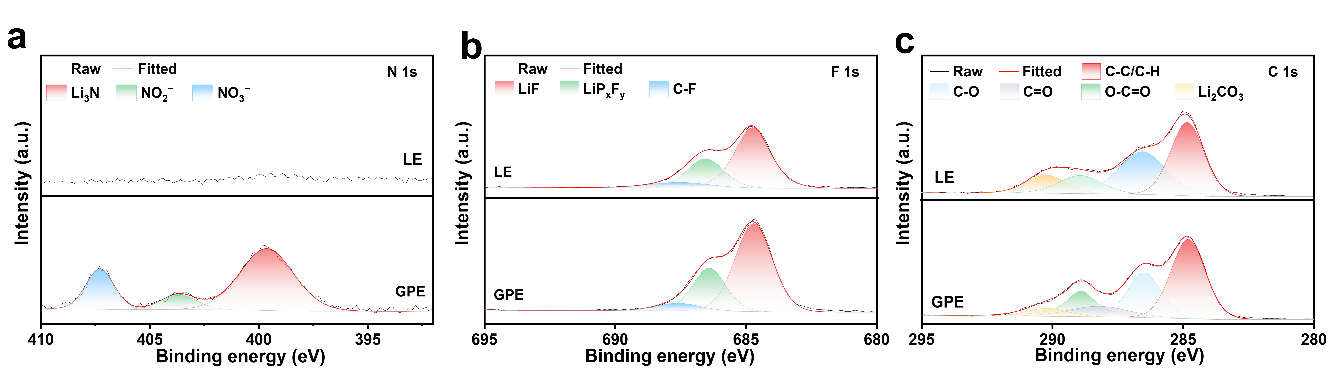
**

**Fig. S16** **a** N 1*s*, **b** F 1*s*, and **c** C 1*s* XPS spectra of the SEI formed in LE and GPE


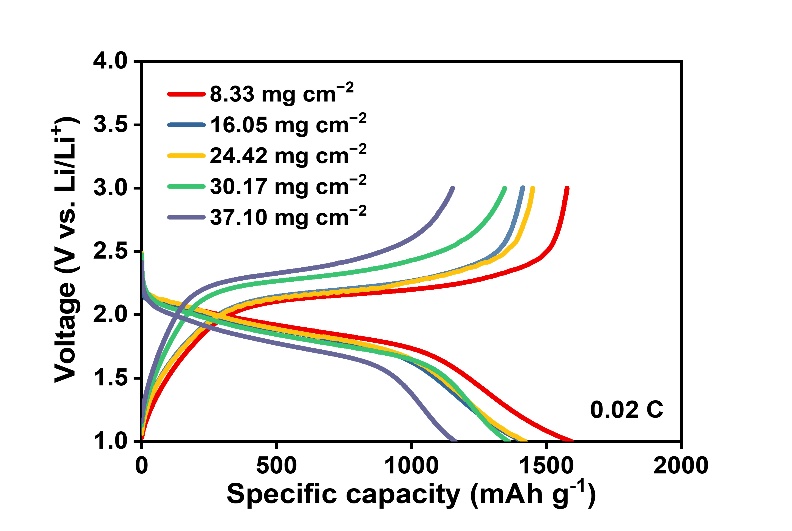


**Fig. S17** Discharge-charge voltage curves of the 3D-HSPAN cathodes with various mass loadings at 0.02 C


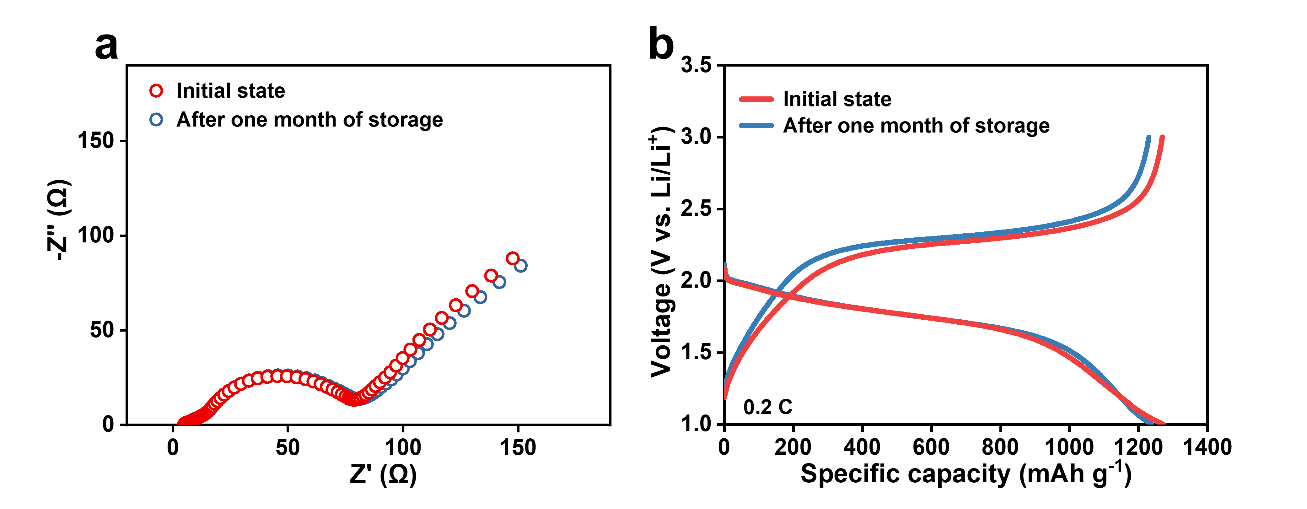


**Fig. S18 a** EIS plots and **b** discharge-charge voltage curves of 3D-HSPAN|GPE|Li before and after one month of storage


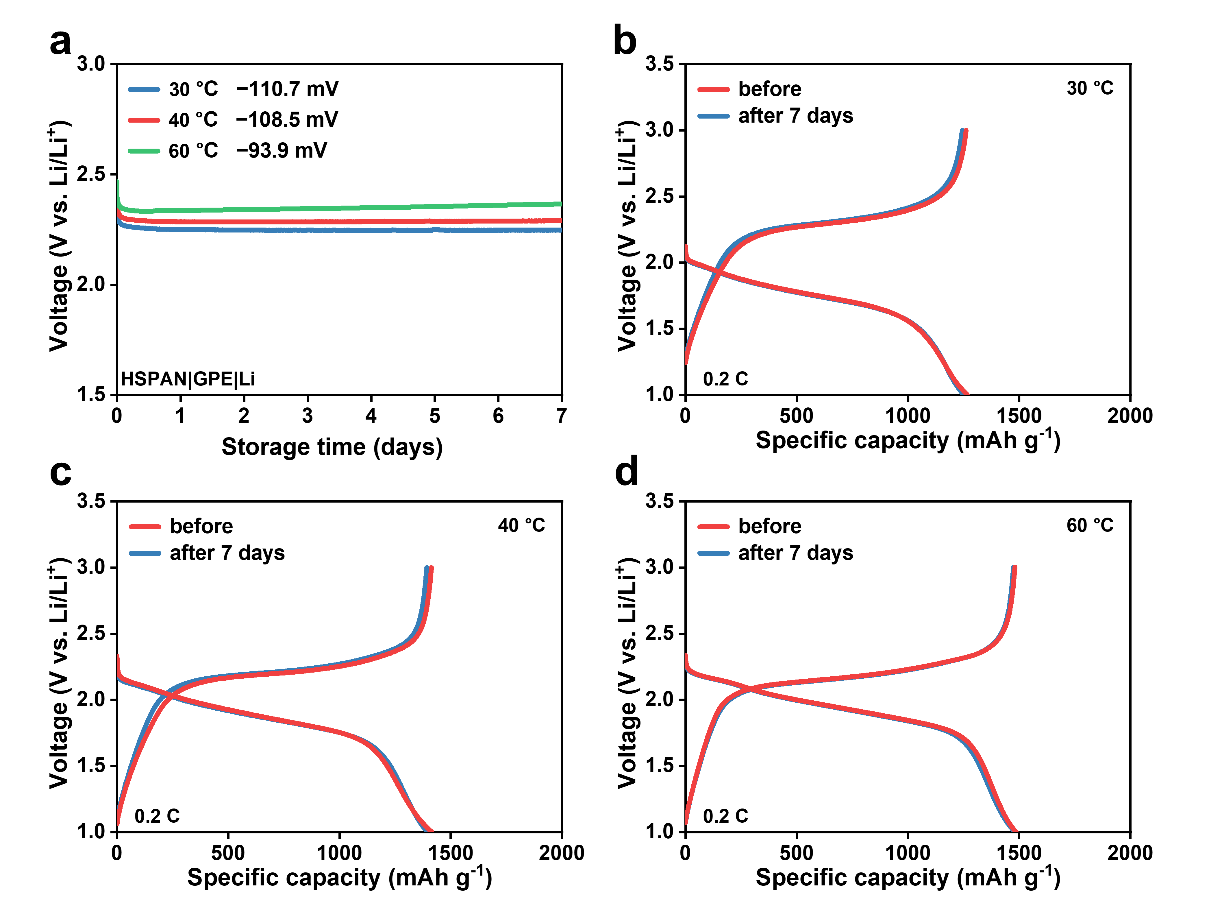


**Fig. S19 a** OCV of 3D-HSPAN|GPE|Li batteries at different temperatures over 7 days of standing. Discharge-charge voltage curves of 3D-HSPAN|GPE|Li batteries before and after 7 days of standing at **b** 30 ^o^C, **c** 40 ^o^C, and **d** 60 ^o^C


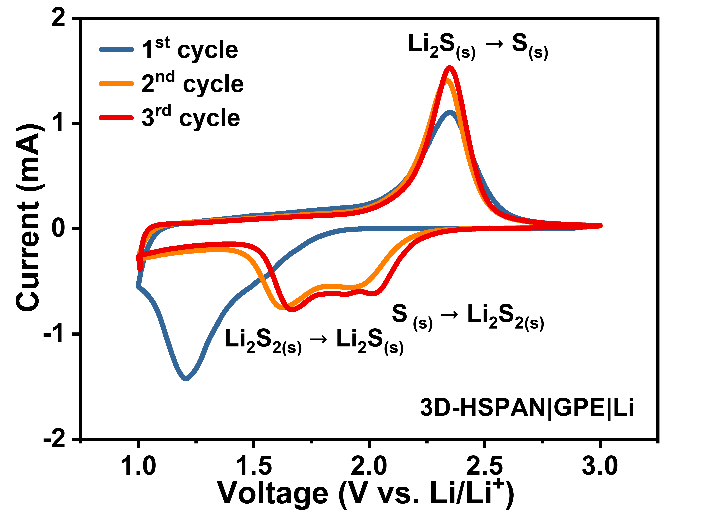


**Fig. S20** CV curves of 3D-HSPAN|GPE|Li cell at a scan rate of 0.1 mV s^–1^

**
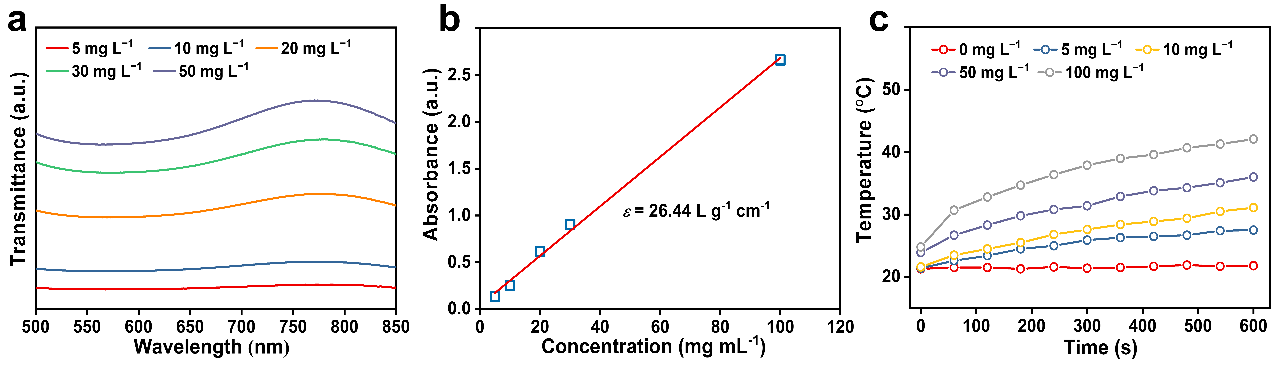
**

**Fig. S21 a** UV-vis-NIR absorption spectra of MXene colloids with different concentrations. **b** Linear fitting of MXene colloid concentration with absorbance in UV-vis-NIR absorption spectra. **c** Photothermal response of the MXene colloid with different concentrations under NIR light at *λ* = 808 nm

**
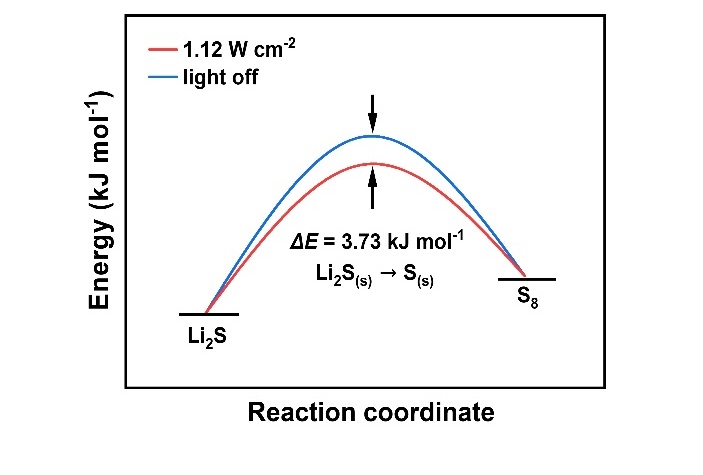
**

**Fig. S22** Relative activation energies for Li_2_S-to-S_8_ conversion

**
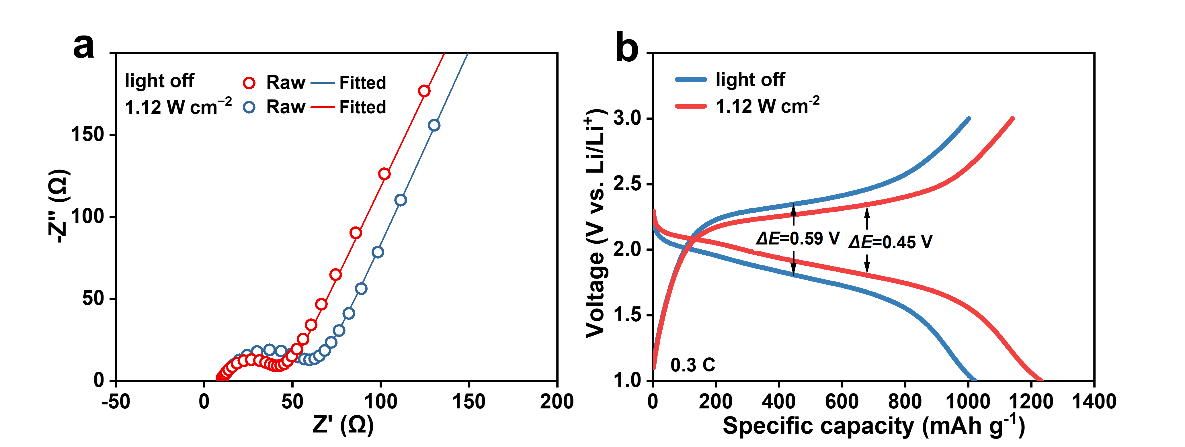
**

**Fig. S23 a** EIS plots and **b** discharge-charge voltage curves of 3D-HSPAN|GPE|Li cells under NIR irradiation or in the dark with 5 wt.% MXene


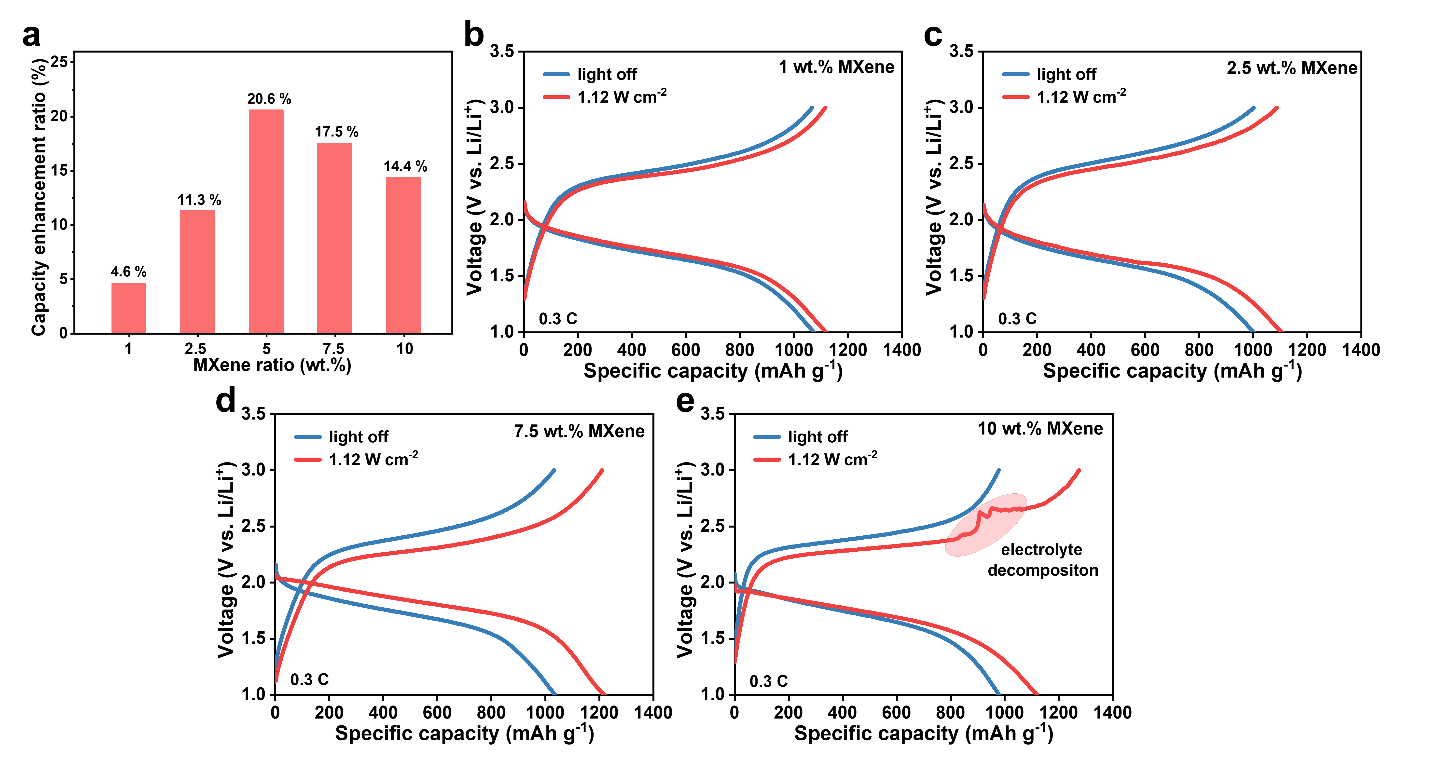


**Fig. S24 a** Capacity enhancement ratio of cathodes with different MXene contents under light illumination. Discharge-charge voltage curves of 3D-HSPAN|GPE|Li cells under NIR irradiation or in the dark with **b** 1 wt.%, **c** 2.5 wt.%, **d** 7.5 wt.%, and **e** 10 wt.%


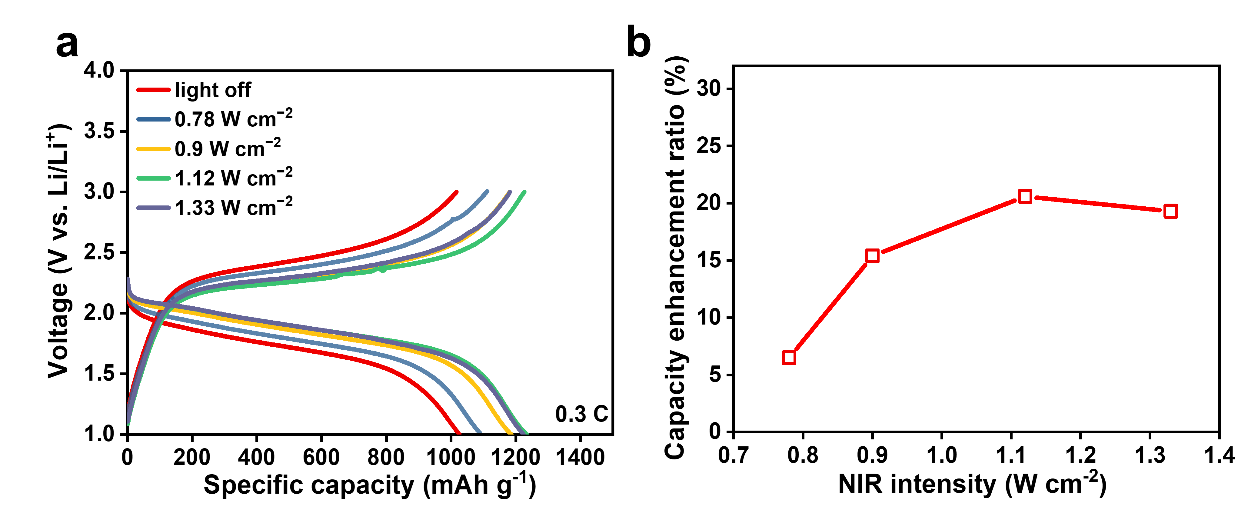


**Fig. S25 a** Discharge-charge voltage curves and **b** capacity enhancement ratio of 3D-HSPAN|GPE|Li batteries at different NIR irradiation powers


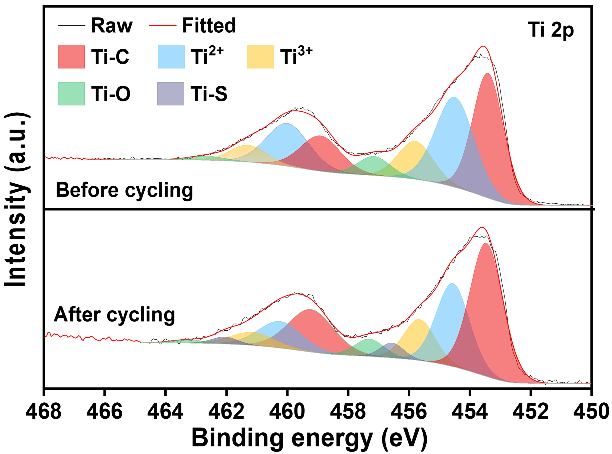


**Fig. S26** Ti 2*p* XPS spectra of the 3D-HSPAN cathode after 45 cycles at 0.3 C under NIR irradiation (1.12 W cm⁻^2^)

**
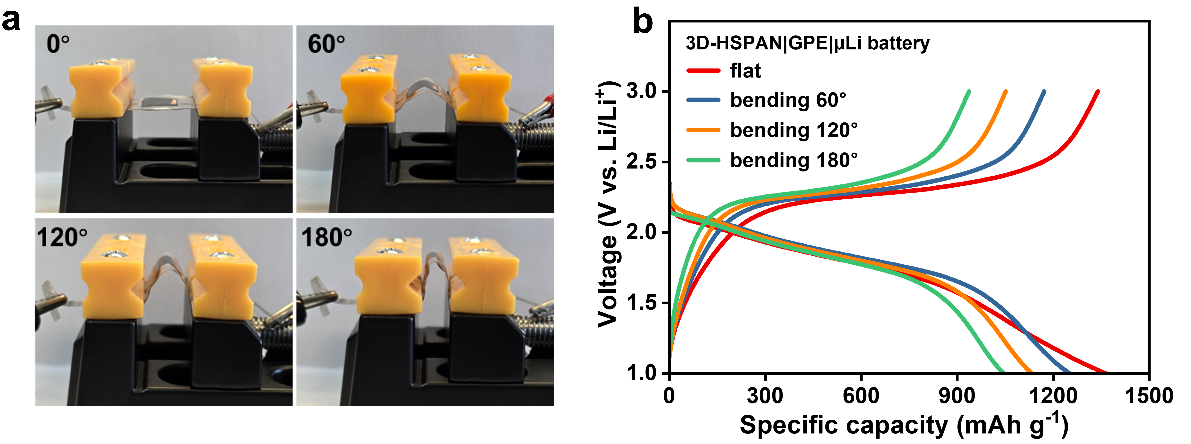
**

**Fig. S27** **a** Optical images of 3D-HSPAN|GPE|μLi micro-batteries at different bending states. **b** Discharge-charge voltage curves of 3D-HSPAN|GPE|μLi micro-battery at different bending states

**Supplementary References**

[S1] Y. Liu, X. Meng, Z. Wang, J. Qiu, A Li_2_S-based all-solid-state battery with high energy and superior safety. Sci. Adv. **8**(1), eabl8390 (2022). <https://www.science.org/doi/10.1126/sciadv.abl8390>

[S2] H. Lin, S. Gao, C. Dai, Y. Chen, J. Shi, A two-dimensional biodegradable niobium carbide (MXene) for photothermal tumor eradication in NIR-I and NIR-II biowindows. J. Am. Chem. Soc. **139**(45), 16235-16247 (2017). <https://doi.org/10.1021/jacs.7b07818>
